# NMR Metabolomics Assessment of Osteogenic Differentiation of Adipose-Tissue-Derived Mesenchymal Stem Cells

**DOI:** 10.1021/acs.jproteome.1c00832

**Published:** 2022-01-21

**Authors:** Daniela S. C. Bispo, Catarina S. H. Jesus, Marlene Correia, Filipa Ferreira, Giulia Bonifazio, Brian J. Goodfellow, Mariana B. Oliveira, João F. Mano, Ana M. Gil

**Affiliations:** † Department of Chemistry, CICECO - Aveiro Institute of Materials (CICECO/UA), University of Aveiro, Campus Universitario de Santiago, 3810-193 Aveiro, Portugal; ‡ Department of Biotechnology Lazzaro Spallanzani, University of Pavia, Corso Str. Nuova, 65, 27100 Pavia PV, Italy

**Keywords:** stem cells, differentiation, osteogenic differentiation, osteogenesis, metabolic switch, NMR, metabolomics, metabonomics

## Abstract

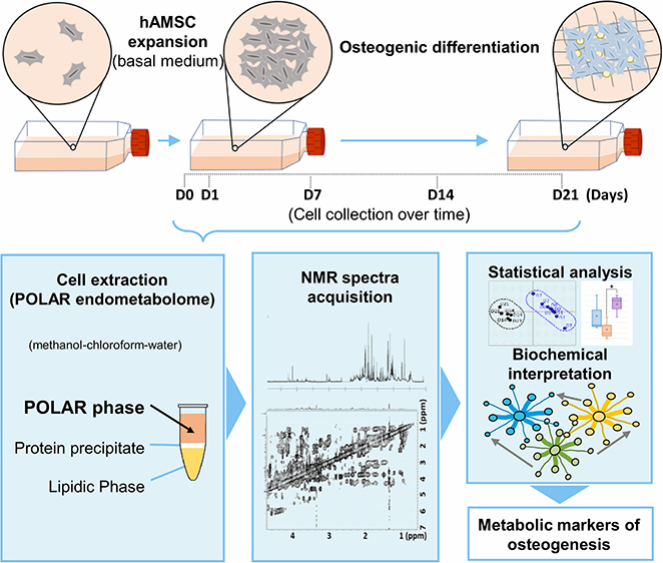

This Article presents, for the first time to our knowledge, an untargeted nuclear magnetic resonance (NMR) metabolomic characterization of the polar intracellular metabolic adaptations of human adipose-derived mesenchymal stem cells during osteogenic differentiation. The use of mesenchymal stem cells (MSCs) for bone regeneration is a promising alternative to conventional bone grafts, and untargeted metabolomics may unveil novel metabolic information on the osteogenic differentiation of MSCs, allowing their behavior to be understood and monitored/guided toward effective therapies. Our results unveiled statistically relevant changes in the levels of just over 30 identified metabolites, illustrating a highly dynamic process with significant variations throughout the whole 21-day period of osteogenic differentiation, mainly involving amino acid metabolism and protein synthesis; energy metabolism and the roles of glycolysis, the tricarboxylic acid cycle, and oxidative phosphorylation; cell membrane metabolism; nucleotide metabolism (including the specific involvement of *O*-glycosylation intermediates and NAD^+^); and metabolic players in protective antioxidative mechanisms (such as glutathione and specific amino acids). Different metabolic stages are proposed and are supported by putative biochemical explanations for the metabolite changes observed. This work lays the groundwork for the use of untargeted NMR metabolomics to find potential metabolic markers of osteogenic differentiation efficacy.

## Introduction

Bone injuries can heal spontaneously through an intricate and well-coordinated process involving several signaling pathways and cell types.^1^ However, the regeneration of large bone defects (caused by trauma, surgical resection, or disease) remains an orthopedic challenge that is significantly enhanced in advanced aging.^2,3^ Engineered bone constructs are promising alternatives to the conventional autologous bone grafts used in clinical applications, potentially overcoming the limited availability of autologous tissue and related clinical complications.^3−5^ In this context, several innovative strategies involving stem cells (SCs, undifferentiated self-renewable cells that have the ability to differentiate into specialized cells) have emerged, in particular, using mesenchymal stem cells (MSCs), which can differentiate into a variety of cell lineages (multipotent), including the osteogenic lineage, thus having large potential in bone regenerative medicine.^6^

Understanding the underlying metabolism of MSC differentiation is of paramount importance for their behavior to be understood and potentially guided toward effective therapies. Untargeted metabolomics is of significant value in this context, as it may unveil novel metabolic information and potential new biomarkers of performance. In a typical metabolomics strategy, analytical data obtained by nuclear magnetic resonance (NMR) spectroscopy or mass spectrometry (MS) for complex mixtures (*e.g.*, biofluids, tissues and cells) are handled and interpreted with the aid of multivariate statistical analysis (MVA).^7,8^ Because local metabolic changes are believed to be critical for tissue regeneration,^9^ metabolomics of MSCs (through cell extracts or fingerprinting and culture media or footprinting) has already provided valuable information on metabolic adaptations associated with differentiation into bone, adipose tissue, or cartilage cells.^10^ Indeed, in recent years, several metabolomic studies have been carried out mostly through MS-based approaches and typically using bone marrow MSCs (BMMSCs).^11−16^ These reports involve the promotion of osteogenic differentiation, either by media supplementation or specific physical properties (biomaterial nanotopography^17,18^ or mechanical stimuli^15,19^), the associated metabolic adaptations having been studied both in traditional *in vitro* conditions^11,12^ and within specific biomaterials, such as nanostructured surfaces^17,18,20−22^ or scaffolds.^19,23,24^ The results suggest that regardless of the osteoinductive method or culture conditions, osteogenic differentiation seems to be consistently associated with a generalized metabolic upregulation, often shown by the initial accumulation of amino acids, carbohydrates, nucleotides, or lipids, among other compounds.^12,16−20^ Some reports suggest a subsequent metabolic reversal toward the end of the process, with differentiated cells acquiring a metabolic profile resembling that of primary osteoblasts.^12,23^ In addition, MSC differentiation seems to lead to unique lineage-specific lipidic profiles, with osteoblastic membrane phenotypes containing longer and more polyunsaturated fatty acids (PUFAs, such as docosahexaenoic acid (DHA)) compared with undifferentiated cells.^13^ Interestingly, specific lipids (*e.g.*, DHA and cholesterol sulfate) have been suggested to have osteoinductive properties.^13,15^ Furthermore, it is known that MSCs isolated from distinct tissue sources often exhibit different proliferation, differentiation, and immunological properties^25,26^ and therefore may be expected to exhibit different metabolic profiles. As previously mentioned, in the case of osteogenic differentiation, the majority of metabolomic studies have addressed MSCs from bone marrow,^11,13−17,19−21,27−29^ adding to only a few publications using umbilical cord MSCs.^12,23,30^ The use of adipose tissue in this context is increasingly interesting, including as a promising source of MSCs capable of osteogenic differentiation, because it is usually considered clinical waste and is typically available in large amounts from minimally invasive clinical procedures.^31^ However, metabolomic studies of adipose-derived MSC (AMSC) differentiation are still scarce, to the best of our knowledge, with only a few recent reports evaluating osteogenic differentiation of AMSCs,^24,32^ including a study on adipose perivascular SCs,^33^ along with some studies of adipogenesis.^34−36^ More specifically, the metabolic impact of different tissue sources of rabbit MSCs (adipose tissue and skeletal muscle) on adipo-osteogenic differentiation has been compared using MS metabolomics of lipidic extracts.^32^ Interestingly, although different lipid profiles were observed toward the end of osteogenic differentiation, depending on the tissue origin, the enrichment of cell membranes in specific *N*-acyl-phosphatidylethanolamine species appeared to be characteristic of osteogenic lineages, regardless of the source. In addition, a combination of liquid chromatography–mass spectrometry (LC-MS) metabolomics and transcriptomics evaluated the effect of a 3D nanocomposite scaffold (nanohydroxyapatite/polyurethane layers with interspersing layers of decellularized bovine bone particles) on the osteogenic differentiation of human AMSCs (hAMSCs) compared with 2D standard cultures.^24^ The results showed that several endometabolome changes were similar in the scaffold and in the 2D culture (namely, regarding the metabolites inosine monophosphate, glycerol-3-phosphate, and 1-methylhistidine). Associated transcriptomics data revealed interactions among bone morphogenetic protein (BMP), Hedgehog, and wingless-related integration site (Wnt) signaling pathways related to the osteogenic potential of the scaffold.

In this work, a detailed metabolomic analysis of the polar endometabolome of hAMSCs was carried out during osteogenic differentiation using untargeted ^1^H NMR metabolomics, for the first time to our knowledge, with the aim of characterizing the dynamic metabolic changes taking place throughout the 21 days of osteoinduction. This work builds on previous MS metabolomics studies that monitored AMSC osteogenic differentiation,^24,32^ exploiting the different and complementary characteristics of NMR^37^ (typically of a holistic nature, high reproducibility, albeit lower sensitivity: submillimolar compared with less than picomolar in MS). The metabolic adaptations identified here pave the way for the potential definition of metabolic biomarkers of the osteogenic differentiation capacity of hAMSCs.

## Experimental Section

### Cell Culture and Osteogenic Differentiation

**Figure 1:**
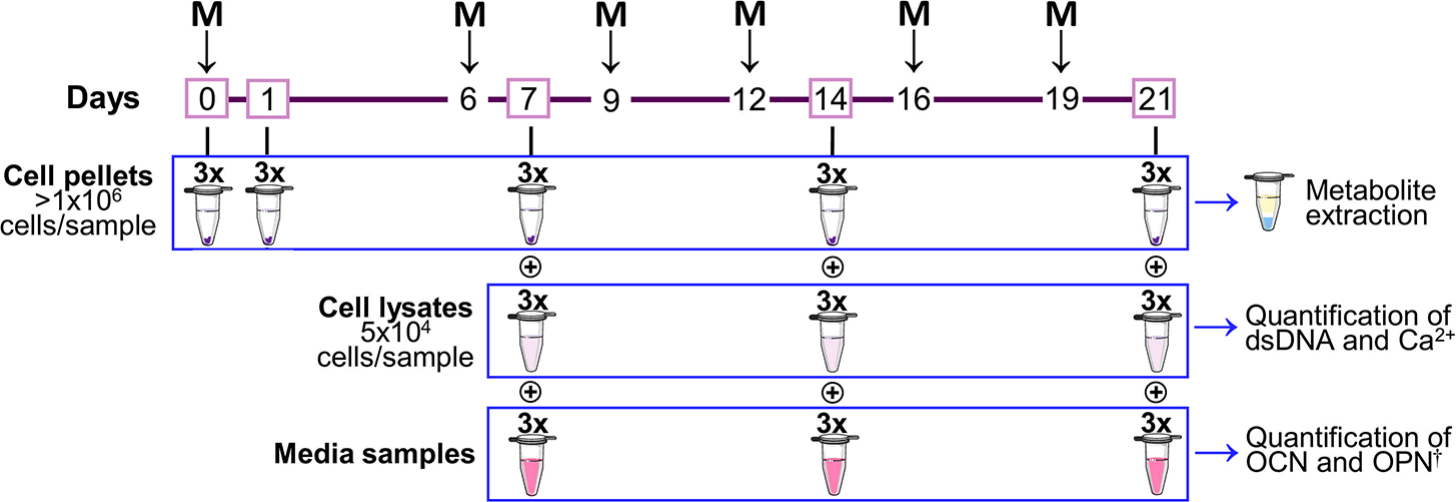
Schematic representation of the osteogenic differentiation timeline (days 0–21) showing the time points where medium exchanges (**M**) and sample collection (day numbers in pink boxes), in triplicate, were carried out. Cell pellets were used for metabolite extraction and subsequent NMR analysis of the polar phase. Cell lysates were used for the dsDNA and calcium evaluation, whereas medium samples were used for the osteogenic markers assessment (namely, OCN and OPN). M, medium exchange; ^†^, OPN measurement was attempted, however, results were too low and variable to be discussed, possibly due to potential high retention of the protein in the extracellular matrix. Abbreviations: dsDNA, double-stranded deoxyribonucleic acid; OCN, osteocalcin; OPN, osteopontin.

Human AMSCs were purchased from the American Type Culture Collection (ATCC PCS-500-011). Cells were thawed, plated in culture flasks (T175) and expanded under basal conditions in minimum essential alpha medium (α-MEM, Gibco 12000063) supplemented with 10% v/v heat-inactivated fetal bovine serum (FBS, Gibco 10270106) and 1% v/v antibiotics (penicillin–streptomycin, Gibco 15240062) at 37 °C in a humidified 5% CO_2_ incubator. When they reached near 100% confluence, cells were thoroughly rinsed with Dulbecco’s phosphate-buffered saline (dPBS, Corning 55-031-PC) and passaged using a 0.25% (v/v) trypsin–EDTA solution (Gibco 27250018) at 37 °C for 5 min. The detachment reaction was stopped by the addition of basal culture medium. For osteogenic differentiation, hAMSCs were harvested and seeded at a density of 0.5 × 10^6^ cells/flask (passage 7). After incubation under the conditions previously described and after confluence levels reached ∼100%, the culture medium was exchanged and supplemented with osteogenic differentiation factors, namely, 10 mM β-glycerophosphate (Sigma-Aldrich G9422), 50 μg/mL l-ascorbic acid (Sigma A0278), and 10 nM dexamethasone (ACROS Organics 230300010). The osteogenic medium was exchanged twice a week throughout 21 days, specifically on days 0, 6, 9, 12, 16, and 19 (Figure 1). Throughout the 21 day period of osteoindunction, cells were trypsinized (as previously described) and collected in triplicate on days 0, 1, 7, 14, and 21 (Figure 1). Cell suspensions were filtered through 100 μm pore strainers, then centrifuged (300*g*, 4 °C, 5 min) and resuspended in phosphate-buffered saline (PBS) twice to avoid medium contamination. On the basis of preliminary experiments with different numbers of hAMSCs and according to previous NMR studies on other SC types,^38^ at least 1 × 10^6^ cells (counted in a Neubauer chamber) per pellet were allocated for metabolomics. These samples were immediately extracted (described below), and the polar phases were stored at −80 °C until analysis. In addition, at least 5 × 10^4^ cells were kept for biochemical testing. These samples were subjected to lysis by osmotic/thermal shock and stored at −80 °C (Figure 1). Prior to biochemical analysis, cell lysates were thawed at room temperature (RT), exposed to ultrasound for 10 min, and kept overnight at −20 °C. This was repeated twice to improve the DNA extraction. Medium samples were also collected, as shown in Figure 1, for osteocalcin (OCN) and osteopontin (OPN) quantification. (However, the results for the latter were too low and variable to be discussed, possibly due to the potential high retention of the protein in the extracellular matrix.)

### Quantification of dsDNA

Cell lysate samples were centrifuged (450*g*, 5 min, RT ∼20 °C), and the supernatant was used for double-stranded DNA (dsDNA) quantification. The Quant-iT PicoGreen dsDNA assay kit (Molecular Probes, Invitrogen) was employed according to the manufacturer’s instructions. The dye fluorescence was measured in a microplate reader (Synergy HTX, Biotek Instruments) at emission and excitation wavelengths of 528 ± 10 nm and 485 ± 10 nm, respectively. For each sample, the dsDNA concentration was calculated using a calibration curve (dsDNA concentration range: 0.0 to 2.0 μg/mL).

### Quantification of Secreted Osteocalcin and Calcium

OCN secretion by hAMSCs was evaluated at days 14 and 21 of osteogenic differentiation using *in vitro* SimpleStep ELISA (enzyme-linked immunoabsorbent assay) kits for human OCN (ab270202, Abcam) and human OPN (ab269374, Abcam), respectively, according to the manufacturer’s instructions. OCN levels were quantified by measuring the absorbance at λ = 450 nm in a microplate reader (Synergy HTX, Biotek Instruments) and were expressed in nanograms of protein, normalized to dsDNA content. (As previously mentioned, OPN quantification produced too low and variable results, hindering their discussion.) Regarding calcium quantification, 20 μL of each sample (cell lysates from days 7, 14, and 21) and each calcium standard (0, 4, 6, 8, and 10.0 mg/dL) was mixed with 20 μL of HCl (1 M) for 30 min at RT in a 96-well plate. In another 96-well plate, 20 μL of the previously prepared solutions were added to 80 μL of the reagents provided in the QuantiChrom calcium assay kit (DICA-500, BioAssay Systems), according to the instructions of the manufacturer. Absorbance readouts were measured at λ = 612 nm using a microplate reader (Synergy HTX, Biotek Instruments). Calcium concentrations were normalized by the total dsDNA in each sample.

### Metabolite Extraction

Intracellular metabolites were extracted using the methanol–chloroform–water method, as described elsewhere.^39^ In brief, cell pellets were resuspended in 800 μL of a cold solution of methanol (Honeywell Riedel-de-Haën 14262) and Milli-Q water (4:1), transferred to Eppendorf tubes containing 150 mg of glass beads (ø = 0.5 mm), and vortexed for 2 min (2500 rpm, RT). Then, 320 μL of cold chloroform (Honeywell Riedel-de-Haën 650471) was added to each sample (vortexed for 2 min, 2500 rpm, RT), followed by 320 μL of cold chloroform and 288 μL of cold Milli-Q water (also vortexed for 2 min at 2500 rpm, RT). After 10 min at −20 °C, samples were centrifuged (15 min, 10 000*g*, 4 °C), and lipophilic and polar phases were separated, although only the latter were used for this work. The polar extracts were dried under vacuum and stored at −80 °C until analysis.

### NMR Spectroscopy

Aqueous extracts were resuspended in 650 μL of 100 mM phosphate buffer at pH 7.4, previously prepared in D_2_O (99.9% deuterium, Eurisotop D216) and 0.1 mM 3-(trimethylsilyl)-propionic-2,2,3,3-*d*_4_ acid (TSP, in D_2_O, Sigma-Aldrich 293040), for chemical shift referencing. After vortex homogenization, 550 μL of solution was transferred to 5 mm NMR tubes. NMR spectra were recorded on a Bruker Avance III spectrometer operating at 500.13 MHz for ^1^H (at 298 K). Standard 1D spectra were acquired with the noesypr1d pulse sequence using a 7002.801 Hz spectral width, 32 k data points, a 2.3 s acquisition time, a 4 s relaxation delay (d1), and 512 scans. Each FID (free induction decay) was zero-filled to 32 k points, multiplied by a 0.3 Hz exponential line-broadening function prior to the Fourier transform. Spectra were manually phased and baseline-corrected, and chemical shifts were referenced internally to TSP at δ 0.00. Peak assignments were based on 2D ^1^H–^1^H total correlation (TOCSY) and 2D ^1^H–^13^C heteronuclear single quantum correlation (HSQC) spectra analysis, spiking experiments, literature, and spectral databases, such as the Bruker BBIOREFCODE AMIX database, the human metabolome database (HMDB),^40^ and Chenomx NMR Suite (Chenomx, Edmonton, Canada).

### Statistical Analysis and Other Spectral Analysis

Multivariate analysis was applied to the full-resolution ^1^H NMR spectra using SIMCA-P 11.5 (Umetrics, Umeå, Sweden), with water (5.11–4.69 ppm) and TSP (0.14–0.00 ppm) excluded from the matrices. Because of contamination, methanol (3.38–3.34 ppm) and ethanol (3.69–3.63 and 1.20–1.17 ppm) were also excluded. Spectra were aligned using recursive segment-wise peak alignment^41^ to minimize chemical shift variations, and data were normalized to the total spectral area to account for sample concentration (*i.e*., cell numbers) differences. Principal component analysis (PCA, unsupervised analysis used to detect intrinsic clusters and outliers within the data set) and partial-least-squares discriminant analysis (PLS-DA, supervised analysis to maximize class discrimination) were performed after centering and unit variance (UV) scalings of the spectra, respectively.^42^ The corresponding loading weights were obtained by multiplying each variable by its standard deviation and were colored according to each variable importance to the projection (VIP) (Matlab R2012a). The relevant peaks were integrated from the original spectra using Amix 3.9.5 (Bruker BioSpin, Rheinstetten, Germany) and normalized to the total spectral area. The individual metabolites that most contributed to class separation were selected based on their statistical significance (*p* values <0.05 in the Wilcoxon rank-sum nonparametric test)^43^ and effect size^44^ (|ES| > 0.5 and ES error <80%). For multiple testing, *p* values were adjusted using the Bonferroni correction.^45^ Considering the normalized integrals of each metabolite, the percentage of variation (%var.) between time points was also calculated. Statistical tests and heatmaps were built using Python 3.6.5. Considering all ^1^H NMR spectra acquired, one-dimensional statistical total correlation spectroscopy (STOCSY)^46^ was carried out in selected cases to aid the assignment of some peaks.

## Results

### Biochemical Evaluation of Osteogenic Differentiation

Figure S1a shows that the amount of Ca^2+^ deposited by hAMSCs (normalized to total dsDNA detected) is equivalent at days 7 and 14 and increases significantly (*p* value <0.05) after day 14 of osteoinduction. This illustrates that, as expected, at the end of differentiation (day 21), cells have significantly higher matrix deposition compared to with previous time points, demonstrating active mineralization involving both inorganic phosphate (Pi) and calcium ions (Ca^2+^) for hydroxyapatite formation in the extracellular matrix.^47,48^ To further evaluate the occurrence of osteogenic differentiation, we assessed the expressions of OCN, a bone *γ-*carboxyglutamic acid matrix protein, and OPN, a secreted phosphoprotein, in medium samples collected at days 14 and 21 (Figure S1b,c). The expression of OCN is only expected to occur after the initial proliferative phase of osteoprogenitors,^48^ and hence OCN was detected at day 14 and reached maximal levels at day 21, confirming the progression of mineralization. Overall, these results confirm the occurrence of osteogenic differentiation in the hAMSCs employed in this work.

### Visual Inspection of ^1^H NMR Spectra of hAMSC Polar Extracts

**Figure 2:**
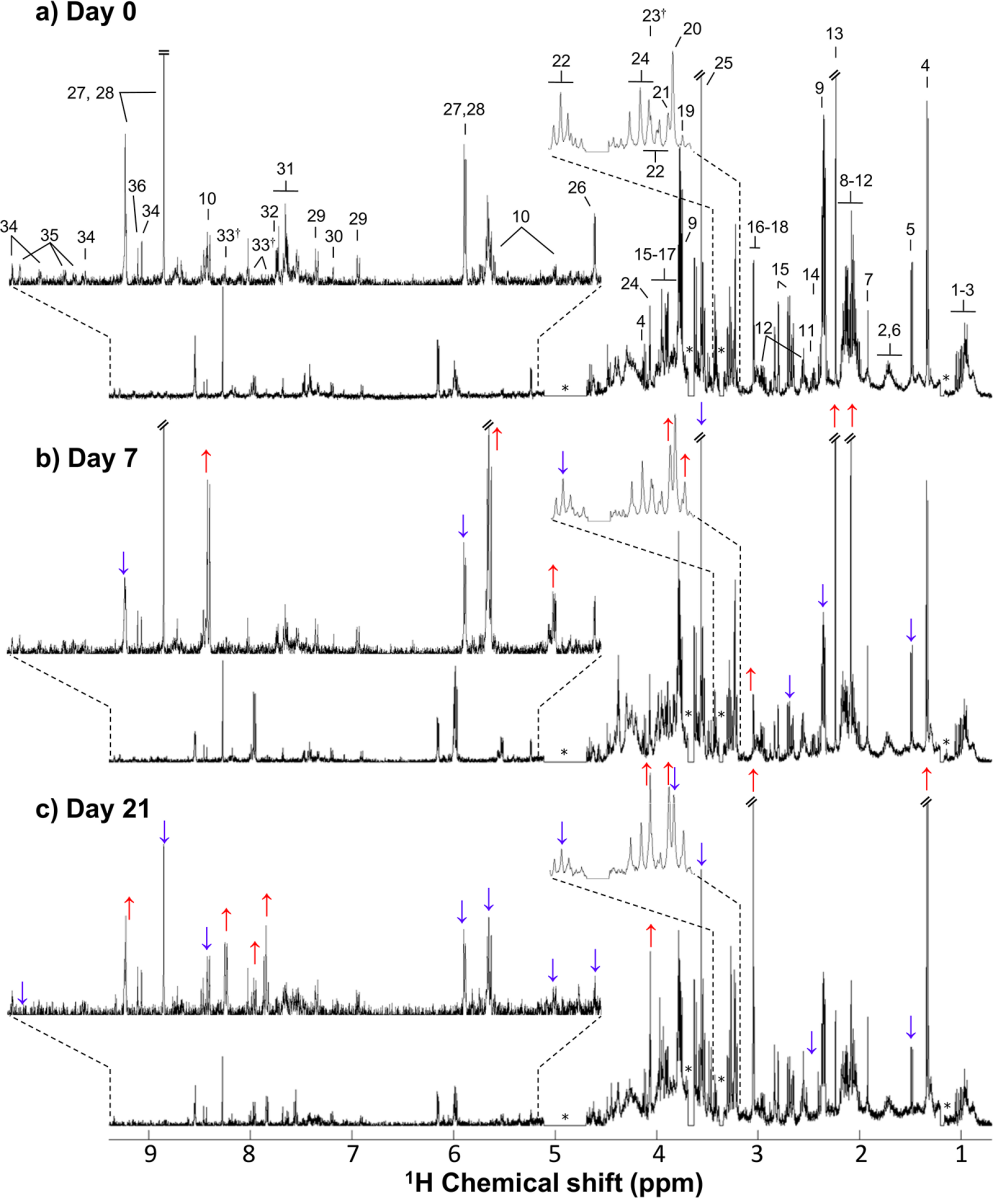
Representative ^1^H NMR spectra (500 MHz) of polar extracts obtained from hAMSC samples collected at (a) day 0, (b) day 7, and (c) day 21 of osteogenic medium exposure. The arrows help to identify apparent variations (blue and red arrows indicating decreases and increases, respectively) in the levels of some metabolites on day 7 versus day 0 (in panel b) and on day 21 versus day 7 (in panel c). *: exclusion of water (δ 5.11–4.69), methanol (δ 3.38–3.34), and ethanol (δ 3.69–3.63 and δ 1.20–1.17) resonances (methanol and ethanol were found as contaminants resulting from the extraction procedure and material cleaning procedures, respectively); ^†^: resonances not observed in the spectra of day 0 samples. Peak assignment: 1. isoleucine, 2. leucine, 3. valine, 4. lactate, 5. alanine, 6. lysine, 7. acetate, 8. proline, 9. glutamate, 10. uridine diphospho-*N*-acetylglucosamine (UDP-GalNAc), 11. glutamine, 12. glutathione (reduced) (GSH), 13. acetone, 14. succinate, 15. aspartate, 16. creatine (Cr), 17. phosphocreatine (PCr), 18. creatinine, 19. choline, 20. phosphocholine (PC), 21. glycerophosphocholine (GPC), 22. taurine, 23. betaine, 24. *myo*-inositol (*m-*Ino), 25. glycine, 26. glucose, 27. adenosine diphosphate (ADP), 28. adenosine triphosphate (ATP), 29. tyrosine, 30. histidine, 31. phenylalanine, 32. phthalate (contamination most likely from plastic vials), 33. hippurate, 34. nicotinamide adenine dinucleotide (NAD^+^), 35. 1-methylnicotinamide (1-MNA), 36. formate. The complete list of assigned signals may be found in the legend of Table S1.

A representative ^1^H NMR spectrum of a polar extract of hAMSCs before differentiation (Figure 2a) reveals a large complexity in all regions of the spectrum, with intense lactate (peaks 4), glycine (peak 25), glutamate (peaks 9), and acetone (peak 13) resonances. A complete assignment list is shown in Table S1, with the identification of 44 metabolites in total, comprising amino acids (16 in total) and derivatives (creatine, phosphocreatine (PCr), creatinine, and reduced glutathione (GSH)), choline and choline-containing compounds (phosphocholine (PC) and glycerophosphocholine (GPC)), nucleotides (adenosine monophosphate (AMP), adenosine diphosphate (ADP), and adenosine triphosphate (ATP)) and derivatives (uridine diphospho-*N*-acetylglucosamine (UDP-GlcNAc) and uridine diphospho-*N*-acetylgalactosamine (UDP-GalNAc)), and organic acids (acetate, citrate, formate, hippurate, lactate, pyruvate, and succinate), among other compounds, including acetone, betaine, 1-methylnicotinamide (1-MNA), dimethylamine (DMA), ethanolamine, *myo*-inositol, nicotinamide adenine dinucleotide (oxidized, NAD^+^), and glucose. This is, to our knowledge, the first high-resolution ^1^H NMR spectrum of a polar endometabolome of hAMSCs, adding to a recent NMR report that compared the exometabolomes of mouse AMSCs harvested from subcutaneous and visceral adipose tissues.^49^ Other reports on AMSC metabolomics were MS-based and compared the endo- and exometabolomes of AMSCs with other MSCs types and studied the impact of donor obesity on hAMSC metabolic profiles.^50−52^ A visual inspection of the spectra in Figure 2 suggests that compared with undifferentiated cells, cells harvested at day 7 of osteogenic differentiation are characterized by increased levels of choline, GPC, and UDP-GlcNAc and decreased levels of glutamate, PCr, and ATP (Figure 2b). Although the timeline of osteogenic differentiation depends on the specific culture conditions^12^ and tissue source of MSCs,^53^ it is generally expected that day 7 is an important turning point in the differentiation process.^48,54,55^ After this point, cells tend to acquire a less proliferative phenotype, enhancing the development/maturation of the extracellular matrix for mineralization. The last day of the process (Figure 2c) is visually distinct from previous time points, with increased levels of citrate, creatinine, betaine, hippurate, and ADP and decreased levels of taurine. However, these qualitative changes may not hold statistical relevance or be representative of the whole sample group, and thus multivariate and univariate statistical analysis are required to confirm/discard apparent visual changes; in any case, most of these were revealed to be significant, with the exception of creatinine and betaine (which showed more variability).

### Statistical Analysis and Relevant Metabolite Changes in Polar Extracts during Osteogenic Differentiation

**Figure 3:**
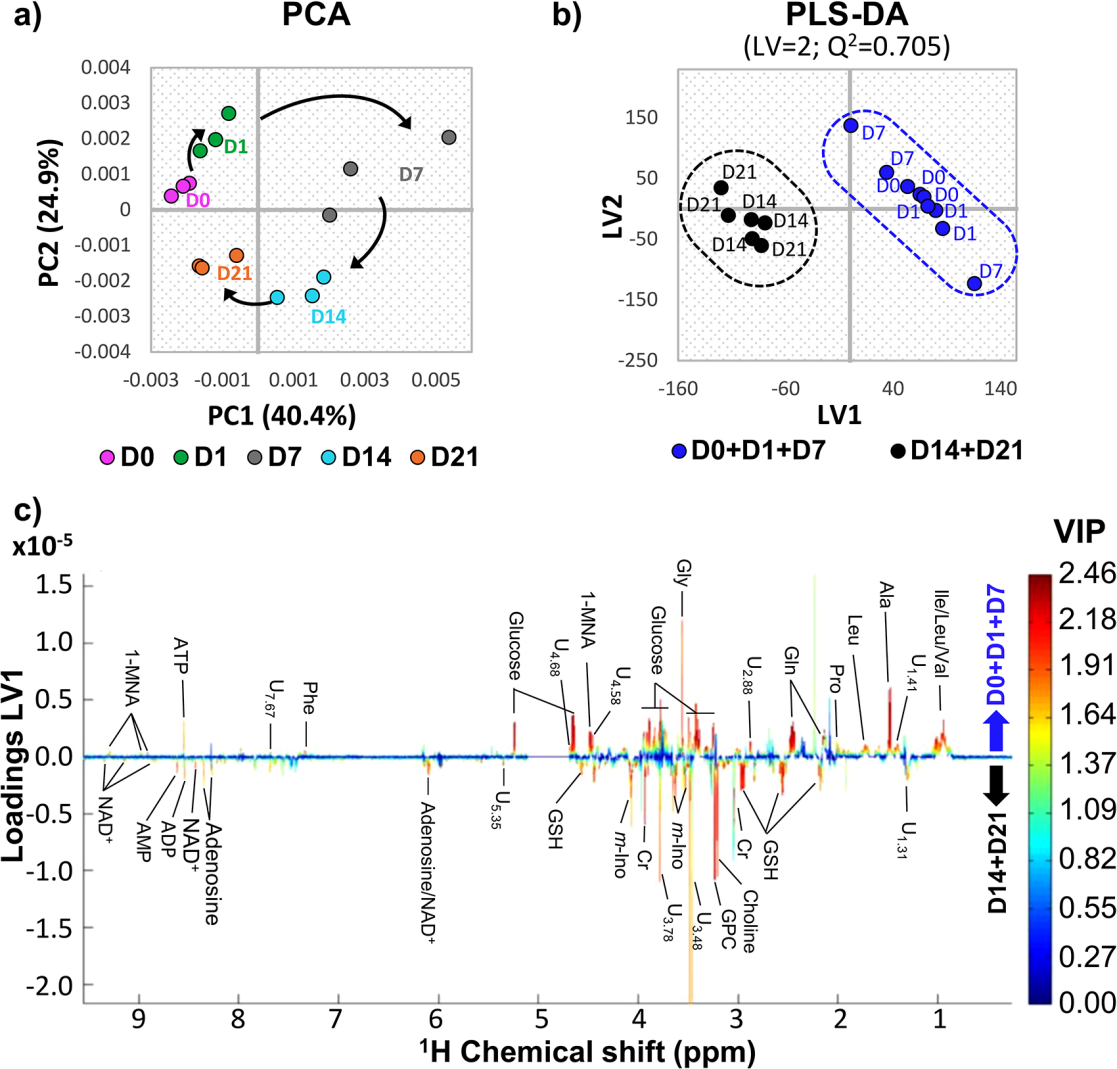
Multivariate analysis results for full-resolution ^1^H NMR spectra of aqueous extracts from hAMSC throughout osteogenic differentiation. Scores scatter plots for (a) principal component analysis (PCA) and (b) partial least-squares discriminant analysis (PLS-DA) comparing the initial days of osteogenic differentiation (day 0 to day 7, blue) with the later days (day 14 to day 21, black). All cell samples were collected in triplicate at days 0, 1, 7, 14, and 21. (c) LV1 loadings plot, colored according to variable importance to the projection (VIP), corresponding to the PLS-DA model shown in panel b. Three-letter code is used for amino acids. Metabolite abbreviations are as defined in Figure 2. LV: latent variables; *Q*^2^: predictive power; U_δ_: unassigned signal at chemical shift δ.

Unsupervised multivariate analysis using PCA (Figure 3a) interestingly shows that changes in the metabolic profile of polar extracts start in the early stages of osteogenesis, then go through a subgroup with larger dispersion (variability) at day 7 and continue to evolve until day 21. These results suggest a continuing metabolic trajectory and illustrate the high biochemical activity that characterizes hAMSCs subjected to osteogenic cues. Curiously, although differentiated cells (day 21) remain separated from undifferentiated cells (day 0) in the PCA scores plot, confirming the expected distinct metabolic profiles, the last day of osteoinduction seems to approach day 0, which suggests that some changes that took place during the process tend to return to basal levels. A subsequent PLS-DA model (Figure S2) considering all time points and samples maximized the discrimination between the days of osteoinduction, with day 7 samples remaining disperse. Several other PLS-DA models considering two classes were evaluated. The inclusion of day 7 either in the initial days group (along with days 0 and 1) (Figure 3b) or in the final days group (along with days 14 and 21) resulted in comparable statistically robust PLS-DA models, with predictive power (*Q*^2^) = 0.70 to 0.80, thus confirming the eventful significance of day 7 in the process but visually masking changes that occur in the steps before and after that time point. Either model was useful in identifying the changing metabolites, and, in the case of the model pictured in Figure 3b, the observed group separation is explained by the corresponding loadings plot (Figure 3c), where high VIP positive peaks relate to metabolites increased before day 7, and negative peaks relate to metabolites increased after day 7.

**Figure 4:**
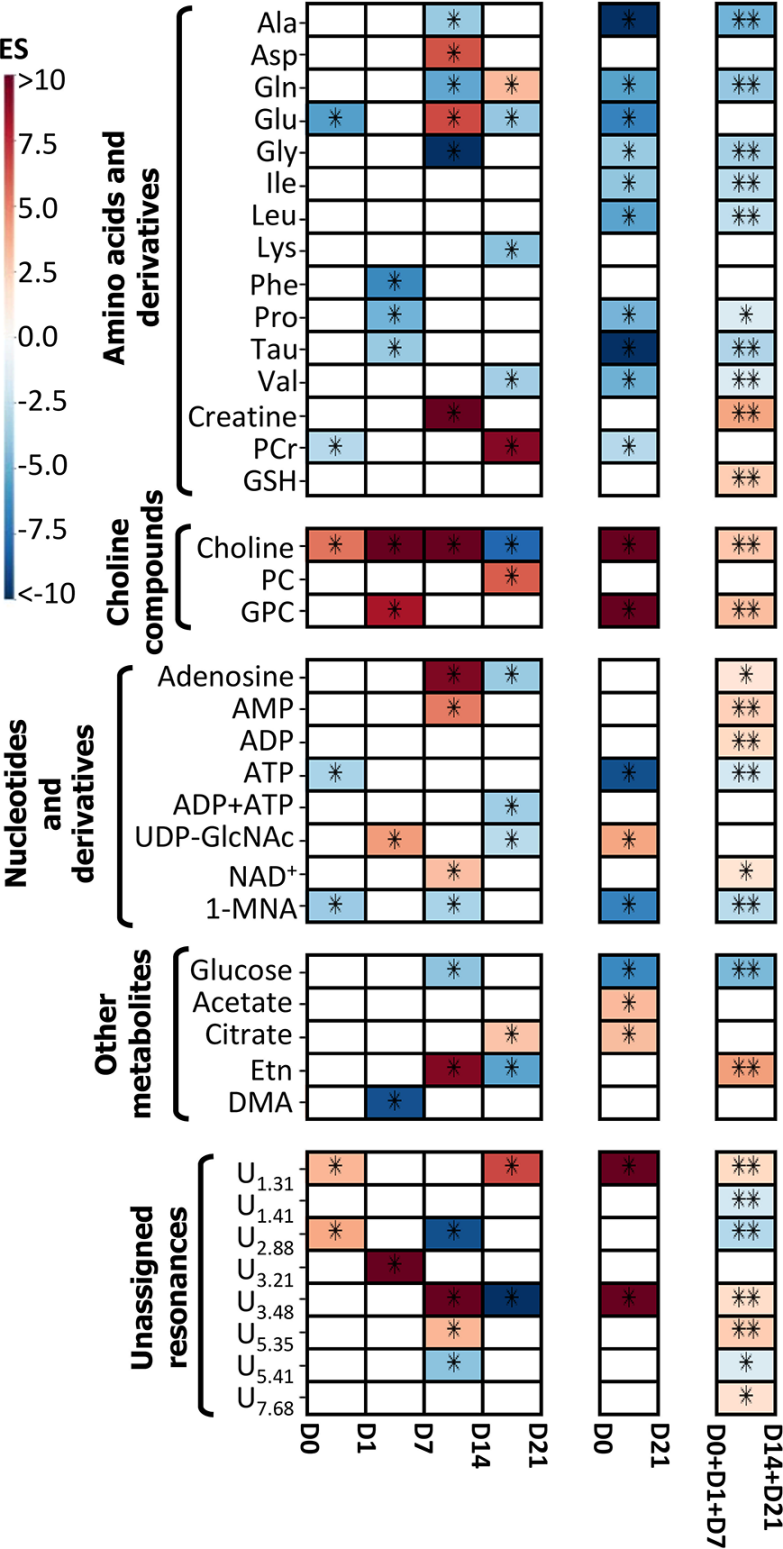
Heatmap displaying statistically significant metabolic variations (|ES|> 0.50, ES error <80%, Wilcoxon rank-sum *p* value <0.05) during the osteogenic differentiation of hAMSCs, shown only in cases where spectral confirmation was observed. Lines represent metabolites, and columns allow for comparisons over time (all time points from day 0 until day 21), between extreme days (day 0 vs day 21, overall balance), and between classes (before day 7 vs after day 7). The color scale varies from minimum (dark blue) to maximum (dark red) effect size (ES) values. Three-letter code is used for amino acids. Abbreviations: 1-MNA, 1-methylnicotinamide; AMP, adenosine monophosphate; ADP, adenosine diphosphate; ATP, adenosine triphosphate; DMA, dimethylamine; Etn, ethanolamine; PCr, phosphocreatine; GPC, glycerophosphocholine; GSH, glutathione (reduced); NAD^+^, nicotinamide adenine dinucleotide (oxidized); PC, phosphocholine; UDP-GlcNAc, uridine diphospho-*N*-acetylglucosamine. U_δ_: unassigned signal at chemical shift δ; *: *p* value <0.05; **: *p* value <0.01.

**Figure 5:**
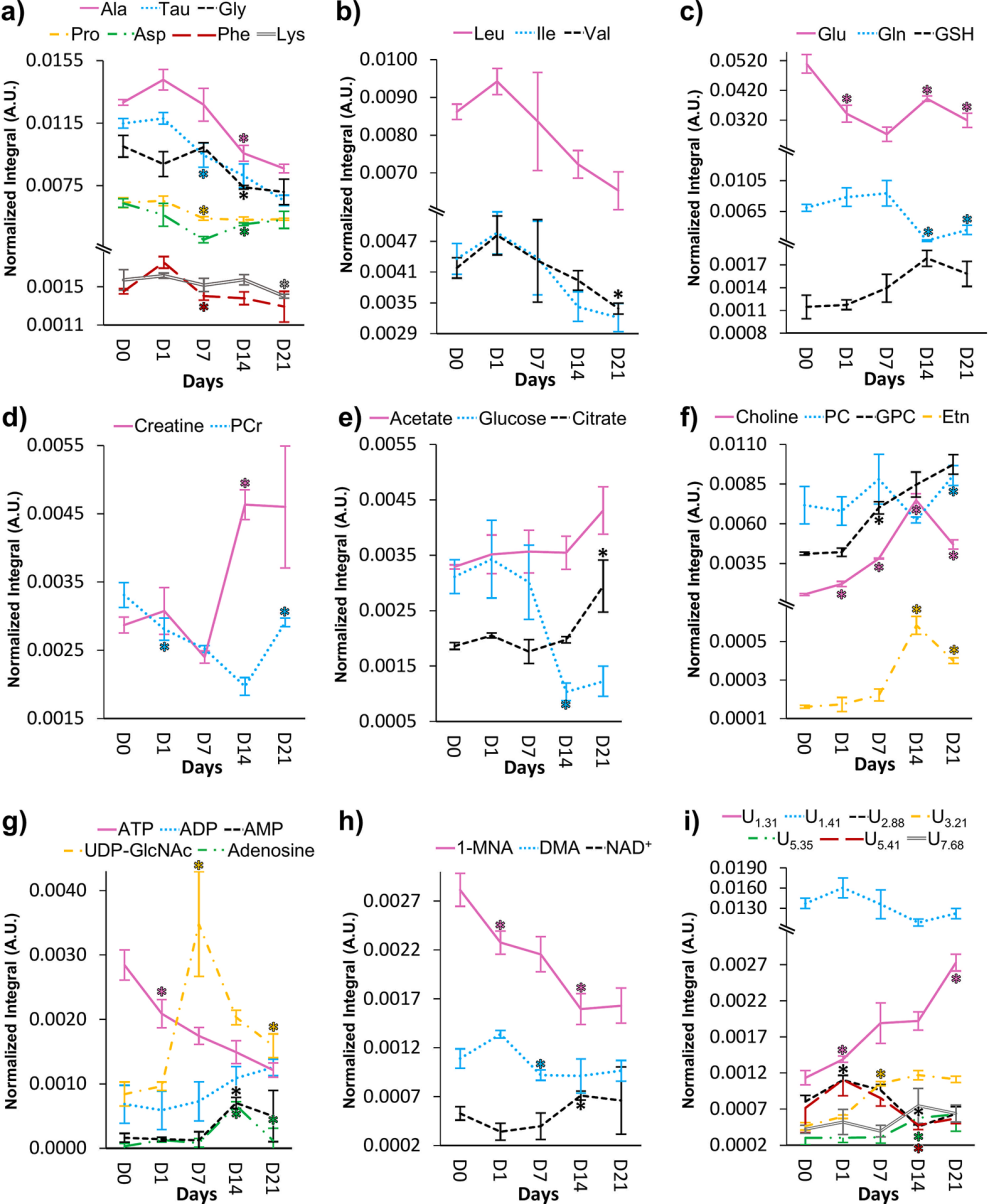
Line graphs displaying the normalized integrals of metabolites over time. (a) Several amino acids, (b) branched-chain amino acids (BCAAs), (c) glutamate, glutamine, and glutathione (GSH), (d) creatine and phosphocreatine (PCr), (e) acetate, citrate, and glucose, (f) choline compounds and ethanolamine (Etn), (g) nucleotides and derivatives (abbreviations as defined in Figure 2), (h) 1-methylnicotinamide (1-MNA), oxidized nicotinamide adenine dinucleotide (NAD^+^), and dimethylamine (DMA), and (i) unassigned signals. Three-letter code is used for amino acids. The statistical significance was calculated (Wilcoxon rank-sum test) between each consecutive time point for each metabolite and is shown only in cases where spectral confirmation was observed, in agreement with Figure 4. *: *p* value <0.05 compared with the previous time point.

Peak integration throughout every step of the process confirmed a larger number of statistically relevant changes along the whole 21 day period (Figure 4 and Table 1), namely, affecting amino acids and derivatives, choline compounds, nucleotides and derivatives, and many other compounds (including still unassigned resonances). By inspecting Table 1, it can be seen that the *p* values exhibit a low statistical value when comparing groups of samples on consecutive days due to the low number of samples per day, yet all changes listed in Table 1 are characterized by *p* values of <0.05, having been confirmed by visual inspection of the spectra. In agreement with the selected PLS-DA model (Figure 3b), higher magnitudes of variation (|ES| > 9.0) were observed between days 7 and 14, specifically for glycine, creatine, choline, adenosine, and an unassigned resonance at 3.48 ppm (Table 1). Of note, all of the remaining changes considered relevant (Table 1) are characterized by |ES| > 1.6. As expected, the comparison of the larger groups of samples collected before and after day 7 (Table S2) produces more statistically relevant *p* values. The most significant changes (filtered for |ES| > 0.50, ES error <80%, and *p* value <0.05) are represented in a heatmap for the sake of clarity (Figure 4). When comparing the early and late stages of differentiation (two right columns in Figure 4), it becomes clear that most varying amino acids (alanine, glutamine, glutamate, glycine, proline, taurine, and the three branched-chain amino acids (BCAAs): leucine, isoleucine, and valine) evolve to net lower levels at the end of osteogenic differentiation, with the exception of creatine and GSH. When analyzing consecutive time points, it is useful to consider the trajectory representations (Figure 5a–d, *: represents only the statistically relevant variations listed in Table 1), where a slight increasing tendency of alanine, phenylalanine, taurine, and BCAAs (Figure 5a,b) is noted between days 0 and 1, and most amino acids decrease thereafter. As shown in the heatmap (Figure 4), phenylalanine, proline, and taurine seem to decrease preferentially and more significantly early on (days 1–7), whereas alanine, glycine, lysine, and valine show significant decreases after day 7. Aspartate, glutamine, glutamate, creatine, and PCr show fluctuations throughout the process (Figures 4 and 5a–d), whereas GSH consistently increases from day 1 and stabilizes at higher values on days 14 and 21 (Figure 5c). Glucose levels exhibit an interesting behavior as they remain relatively high and stable until day 7, after which a marked decrease occurs between days 7 and 14, with levels stabilizing until day 21 (Figures 4 and 5e). This is accompanied by increases in acetate and mainly citrate after day 14 (Figure 5e), which, together with the previously mentioned concomitant changes in PCr and creatine, suggest important adaptations of the energetic metabolism during osteogenic differentiation. Membrane metabolism is also observed to change during osteoinduction, as seen via changes mainly in free choline but also in PC and GPC (Figures 4 and 5). Choline is the only metabolite varying significantly throughout the whole osteogenic period, increasing from early on to a maximum level at day 14 followed by an abrupt decrease at day 21 (Figure 5f). Whereas PC levels are affected by large variability, GPC increases steadily from day 1 until day 21 (Figure 5f). Interestingly, ethanolamine (another membrane lipids precursor) follows the choline trajectory very closely (although at lower levels), reaching maximum levels at day 14. Changes in nucleotide and derivative contents are also of note, with osteogenic differentiation largely affecting the adenosine and uridine metabolism (Figure 5g). Whereas ATP consistently decreases throughout osteoinduction, ADP, AMP, and adenosine tend to increase after day 7 (although adenosine decreases to the initial levels at day 21). Notably, a marked increase was observed in UDP-GlcNAc levels between days 1 and 7 followed by a marked decrease. Other important metabolites varying throughout differentiation include DMA, which decreases between days 1 and 7, NAD^+^ (which, however, does not feature as a main varying metabolite in Figure 4 due to the filtering conditions applied to ES), and 1-MNA. The latter two compounds seem to exhibit almost mirrored behaviors, decreasing and increasing before day 14, respectively. Finally, the fact that PCA showed days 0 and 21 in relatively close proximity (Figure 3a) is probably due to a tendency for some metabolites to return to levels close to those of undifferentiated cells, namely, for adenosine, AMP, DMA, ethanolamine, and PCr (Figures 4 and 5). Conversely, the strong depletion of most amino acids, ATP, glucose, and 1-MNA and the accumulation of acetate, citrate, GPC, and UDP-GlcNAc remain as important distinguishing features between differentiated and undifferentiated hAMSCs. Finally, some unassigned resonances also contribute to the separation of samples in PCA and in PLS-DA, as viewed by the trajectories of peaks resonating at δ 1.31, 2.88, 3.21, and 5.41 (possibly a mono- or oligosaccharide) (Figure 5i).

**Table 1:** Main Statistically Significant Metabolic Differences (Meeting the Limiting Criteria: |ES| > 0.50, ES Error <80%, and Wilcoxon Rank-Sum Test *p* Value <0.05) during Osteogenic Differentiation of hAMSCs Comparing Consecutive Time Points (from Day 0 until Day 21)

		Effect size (ES error, %)^c^			
metabolitemetabolite^a^	*δ*^1^H in ppm (multiplicity)^b^	D_0_ vs D_1_	D_1_ vs D_7_	D_7_ vs D_14_	D_14_ vs D_21_
Amino Acids and Derivatives					
Ala	1.48 (d)			–3.8(70.8)	
Asp	2.82 (dd)			6.4(61.9)	
Gln	2.46 (m)			–5.3(64.3)	3.3(74.6)
Glu	2.35 (m)	–5.6(63.4)		6.6(61.6)	–4.0(69.5)
Gly	3.56 (s)			–11.9(58.2)	
Lys	3.03 (t)				–4.2(68.2)
Phe	7.34 (m)		–6.6(61.6)		
Pro	2.04 (m)		–4.8(65.7)		
Tau	3.42 (t)		–3.9(70.2)		
Val	1.05 (d)				–3.6(72.2)
Creatine	3.04 (s)			13.5(57.8)	
PCr	3.95 (s)	–2.90(79.0)			9.2(59.2)
Choline Compounds					
Choline	3.21 (s)	5.5(63.7)	14(57.7)	12.9(57.9)	–7.9(60.1)
PC	3.23 (s)				6.1(62.4)
GPC	3.24 (s)		8.3(59.8)		
Nucleotides and Derivatives					
Adenosine	8.27 (s)			9.4(59.1)	–3.8(70.4)
AMP	8.62 (s)			5.2(64.4)	
ATP	8.55 (s)	–3.3(74.1)			
ADP+ATP	8.28 (s)				–3.6(71.7)
UDP-GlcNAc	5.52 (dd)		4.4(67.4)		–2.9(79.4)
**NAD**^**+**^	9.15 (d)			3.1(76.6)	
1-MNA	4.49 (s)	–3.7(71.3)		–3.3(74.3)	
Other Metabolites					
Glucose	5.24 (d)			–4.1(68.9)	
Citrate	2.66 (d)				2.9(79.0)
Ethanolamine	3.14 (t)			9.2(59.2)	–5.5(63.7)
Dimethylamine	2.73 (s)		–8.8(59.5)		
Unassigned Compounds					
U_1.31_	1.31 (br)	3.4(73.7)			6.7(61.5)
U_2.88_	2.88 (s)	3.9(69.8)		–8.9(59.4)	
U_3.21_	3.21 (s)		16.4(57.4)		
U_3.48_	3.48 (d)			11.3(58.3)	–12.3(58)
U_5.35_	5.35 (br)			3.4(73.7)	
U_5.41_	5.41 (br)			–4.1(68.5)	

a Three-letter code is used for amino acids. Abbreviations: 1-MNA, 1-methylnicotinamide; AMP, adenosine monophosphate; ADP, adenosine diphosphate; ATP, adenosine triphosphate; GPC, glycerophosphocholine; UDP-GlcNAc, uridine diphospho-*N*-acetylglucosamine.

b Peak used for integration (part of the spin system). Resonance multiplicity: d, doublet; dd, doublet of doublets; m, multiplet; t, triplet; s, singlet; br, broad resonance.

c Effect size (ES) was calculated according to ref (43). (Positive and negative ES values correspond to metabolite level increases and decreases on the second day, compared with the first day, for each comparison.) D_*i*_, day i; U_δ_, unassigned signal at chemical shift δ. Please note that although all *p* values were <0.05, they have low statistical bearing when comparing groups of samples on consecutive days due to the low number of samples per day, yet all changes listed have been confirmed by visual inspection of the spectra.

## Discussion

**Figure 6:**
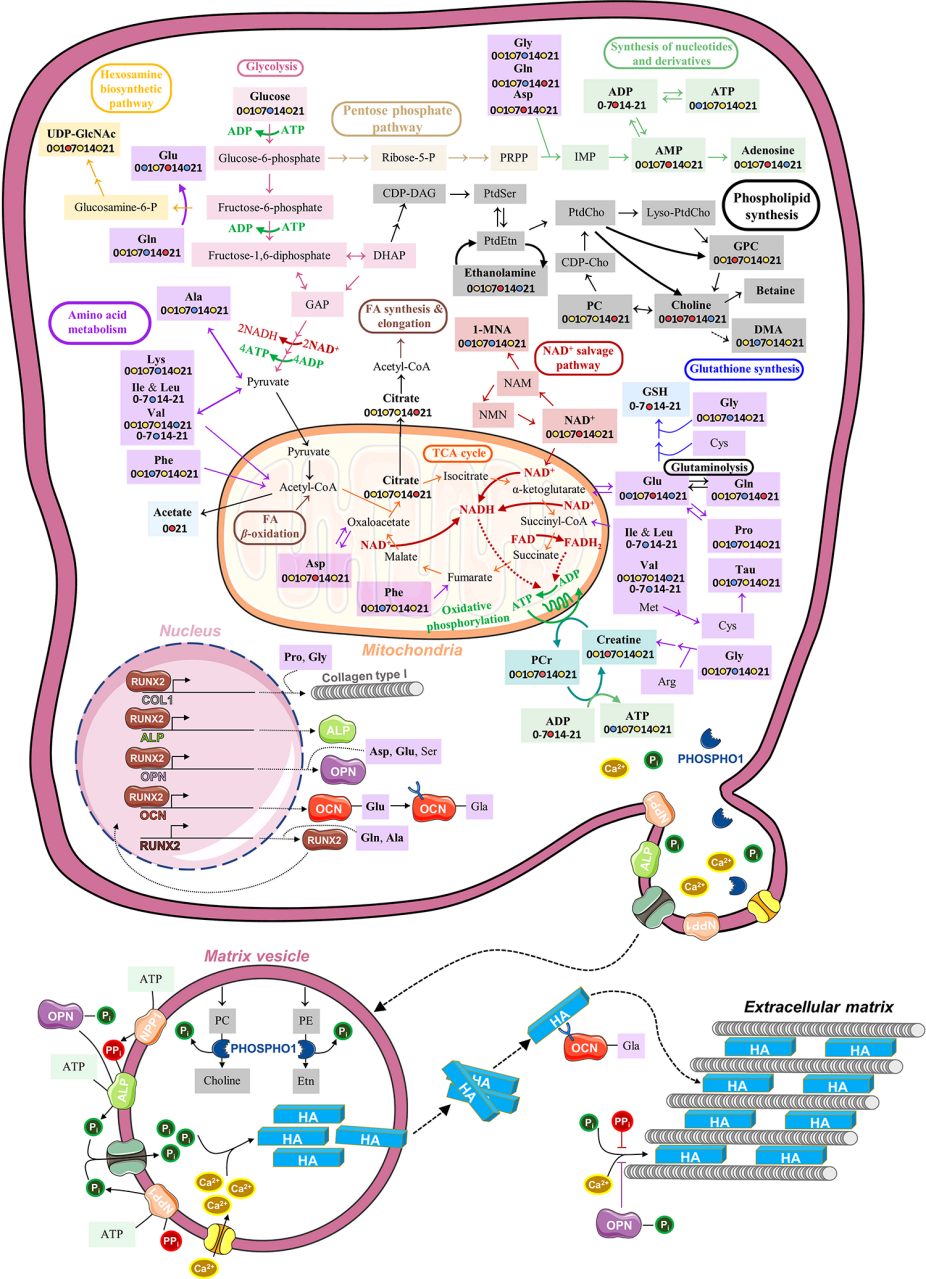
Schematic representation of the main metabolic fluctuations observed in this work throughout the osteogenic differentiation of hAMSCs, along with a simplified depiction of the vesicle-mediated mineralization. Metabolite names in bold indicate compounds identified (additional detected metabolites may be found in Table S1), whereas those that were observed to vary carry a schematic scale as follows: unchanged metabolic levels are represented in yellow, increases are in red, and decreases are in blue. In some cases, an indication is given if the metabolite was seen to vary only between day 0 and day 21 or before and after day 7. Three-letter code is used for amino acids. Abbreviations: ALP, alkaline phosphatase; CDP, cytidine diphosphate; CoA, coenzyme A; DAG, diacylglycerol; DHAP, dihydroxyacetone phosphate; FA, fatty acid; FAD, flavin adenine dinucleotide (oxidized); FADH2, flavin adenine dinucleotide (reduced); GAP, glyceraldehyde 3-phosphate; Gla, *γ-*carboxyglutamate residues; HA, calcium hydroxyapatite; IMP, inosine monophosphate; NADH, nicotinamide adenine dinucleotide (reduced); PRPP, phosphoribosyl pyrophosphate; NPP1, ectonucleotide pyrophosphatase/phosphodiesterase 1; OCN, osteocalcin; OPN, ostepontin; PHOSPHO1, phosphoethanolamine/phosphocholine phosphatase; Pi, inorganic phosphate; PPi, inorganic pyrophosphate; PtdCho, phosphatidylcholine; PtdEtn, phosphatidylethanolamine; PtdSer, phosphatidylserine. Other metabolites are abbreviated as shown in Figure 4. Some elements of this picture were adapted from Servier Medical Art (smart.servier.com) and are licensed under a Creative Commons Attribution 3.0 Unported (CC BY 3.0) license.

In this work, untargeted ^1^H NMR metabolomics was applied, for the first time to our knowledge, to investigate the polar endometabolome of hAMSCs throughout 21 days of osteogenic differentiation. The main metabolic fluctuations identified and their possible association with biochemical pathways are depicted in Figures 5 and 6, respectively. In agreement with previous reports on MSCs from other sources (bone marrow and umbilical cord),^11,12,15,16,28^ the osteogenic differentiation of hAMSCs was demonstrated to be a highly dynamic process. Indeed, in addition to an expected important metabolic switch identified at day 7, significant variations were also found throughout every step of the whole 21-day period, mainly impacting the metabolism of amino acids, energy metabolism, membrane metabolism, nucleotide metabolism, and metabolites involved in antioxidative defense mechanisms.

### Amino Acids and Protein Synthesis

Protein synthesis is essential for the complex process of osteogenic differentiation; therefore, amino acid availability is critical.^48,56^ Although the more significant early variation (from day 0 to day 1) involves a marked decrease in glutamate (%var. = −32.9%), trajectory representations show a tendency for an initial increase in several amino acids over the same period, in particular, for alanine (%var. = +11.4%), phenylalanine (%var. = +20.6%), and the three BCAAs (%var. = +9.3 to +15.2%). Reported studies have, in fact, unveiled an increase in amino acid pools by day 7 of osteogenic differentiation,^12,20^ in preparation for subsequent enhanced protein synthesis^48,57,58^ accompanied by amino acid depletion.^12^ Our results show that in the osteogenic differentiation of hAMSC, this amino acid enrichment does not extend beyond day 7, and processes occurring between days 1 and 7 seem to require phenylalanine (%var. = −19.9%), proline (%var. = −17.2%), and taurine (%var. = −20.0%) to a larger extent (compared to other amino acids), which is followed by a decrease in most amino acids until day 21. Exceptions to a steady decrease include aspartate, glutamate, and glutamine (and creatine and PCr), which may be indicative of the involvement of these particular amino acids in additional processes besides protein synthesis. Regarding protein synthesis, the uptake of glycine (%var._D7→D14_ = −25.8%) and proline (%var._D1→D7_ = −17.2%) residues around day 7 may indicate the onset of type I collagen synthesis, the major organic component of the bone extracellular matrix, which is particularly rich in these two amino acids,^59^ and significant changes in the proline metabolism have also been reported during BMMSC osteogenic differentiation by recent LC-MS metabolomic studies.^16,21^ In addition, Runt-Related Transcription Factor 2 (RUNX2) protein (an activator of the expression of collagen and noncollagenous proteins,^60,61^*e.g.*, alkaline phosphatase (ALP), OCN, and OPN), expected to reach maximum levels close to day 7,^28^ is known to include a domain particularly rich in glutamine and alanine.^60,61^ This may contribute to the decrease observed in both of these amino acids (%var._D7→D14_ = −24.5% for alanine and %var._D7→D14_ = −69.1% for glutamine) around the same time (Figure 4), although both are also expected to feed into the tricarboxylic acid (TCA) cycle (Figure 6). Regarding noncollagenous proteins, OCN and OPN may be particularly important in contributing to specific amino acid depletion, namely, glutamate (%var._D0→D7_ = −36.6%) and aspartate (%var._D0→D7_ = −46.5%). The action of OCN (detected later in the process, as observed here for days 14–21, Figure S1b) involves γ-carboxylation of its glutamate residues, playing an important role in directing the parallel alignment of calcium hydroxyapatite with collagen fibrils (Figure 6).^62^ In addition, OPN (usually expected to work as a later marker^28,48^) is rich in both glutamate and aspartate,^58^ and its increased biosynthesis possibly contributes to glutamate depletion between days 14 and 21 (%var. = −18.7%). This protein provides Pi for hydroxyapatite crystal growth (Figure 6) when dephosphorylated (whereas the phosphorylated form inhibits mineralization and promotes osteoclastic bone resorption instead).^63,64^

Many amino acids may also be used as anaplerotic intermediates for the TCA cycle and in several other metabolic pathways, including hexosamine and nucleotide biosynthesis (Figure 6). This could explain the marked decreases in alanine (%var._D0→D21_ = −33.0%), BCAAs (%var._D0→D21_ = −26.3 to −19.0%), and glutamate (%var._D0→D21_ = −37.4%), with the latter arising from an activation of glutaminolysis, when required. Indeed, glutaminolysis seems to be particularly active between days 7 and 14, as a marked glutamine decrease (%var. = −69.1%) mirrors a simultaneous glutamate increase (%var. = +43.9%), possibly to replenish glutamate levels. The process seems to slow down between days 14 and 21 (Figure 5c), as glutamine increases again (%var. = +50.5%) and glutamate decreases (%var. = −18.7%). Indeed, glutamine (and glutaminolysis) has been recognized before as essential for osteogenic differentiation,^65,66^ contributing to TCA anaplerosis, amino acid biosynthesis (namely, of proline, aspartate, and alanine), and GSH biosynthesis.^66^ Conversely, reduced glutaminolysis activity, in parallel with lower levels of TCA cycle and glycolysis intermediates, has been suggested to reflect a less proliferative metabolic phenotype toward the end of differentiation.^19^ The reduced glutaminolysis activity noted here after day 14 may thus be an indication of less cell proliferation; however, in our work, citrate was the only TCA intermediate seen to vary (increase, %var._D14→D21_ = +49.2%), which, in tandem with the decrease in anaplerotic amino acids, may suggest some enhancement in TCA activity. However, a recent study has proposed glutaminolysis as an important contributor to citrate accumulation, which offers an alternative putative explanation for the increased citrate levels.^67^

In addition to protein synthesis and anaplerotic TCA cycle regulation, some amino acids may be involved in additional specific roles in the osteogenic differentiation of MSCs.^14,16,68,69^ These include the lowering of the essential amino acid lysine (%var._D14→D21_ = −11.0%) toward the end of osteogenic differentiation (as observed here, Figure 4) through its degradation into products, such as saccharopine and l-2-aminoadipate, as seen in the osteogenic differentiation of immortalized BMMSCs.^16^ Also, some amino acids may be linked to antioxidative protection mechanisms; for instance, phenylalanine metabolism seems to be regulated by astaxanthin (an antioxidant carotenoid pigment functioning as an osteogenic differentiation promoter), as observed in rat BMMSCs by LC-MS metabolomics and metabolic pathway enrichment studies,^14^ whereas taurine (seen here to be used up from day 1, Figure 5a) is a recognized player in the protection of osteoblasts against oxidative damage, promoting osteogenic differentiation via the Wnt/β-catenin-mediated activation of the extracellular signal-regulated kinase (ERK) signaling pathway,^68^ consequently increasing the bone mineral density.^69^

### Energy Metabolism

Several studies have suggested that undifferentiated MSCs exhibit low mitochondrial activity relying on glycolysis as their primary energy source, whereas when osteogenic differentiation occurs, oxidative phosphorylation (OxPhos) seems to be activated.^70−72^ Interestingly, mitochondrial OxPhos activation alone has been found to induce osteogenic differentiation via β-catenin acetylation.^72^ In fact, a coordinated regulation between mitochondrial biogenesis and antioxidant protection has been proposed to avoid the accumulation of reactive oxygen species (ROS) when aerobic mitochondrial metabolism becomes dominant in osteogenic differentiation.^70^ In this context, hypoxia-inducible factor 1 (HIF-1, a heterodimeric transcription factor) was suggested as a key regulator of bioenergetic changes during osteogenic differentiation, even in the presence of normal oxygen levels, contributing to the balance between glycolysis and OxPhos.^73^ However, reported data on glycolytic activity still hold some degree of uncertainty, as some studies have noted that glycolysis downregulation occurs during osteogenic differentiation (as viewed by decreasing levels of lactate and glycolytic enzymes),^70,71^ whereas others indicate that differentiating MSCs maintain similar glycolytic activity to that of undifferentiated cells and that OxPhos becomes relatively more active when cell proliferation is less pronounced, at the end of osteogenic differentiation.^73^ (It is important to note, however, that such effects may depend on the type of MSCs under study.) Our results show that glucose levels in differentiating hAMSCs remain stable until day 7 (as do lactate levels, which do not vary throughout the whole 21-day period), undergoing a sudden marked decrease between days 7 and 14 (Figure 5e, %var. = −65.7%) and then remaining low and stable until day 21. We suggest that this indicates an enhancement in glycolysis activity between days 7 and 14, which could result in some increased activity of the TCA cycle and OxPhos. However, this did not result in significant TCA intermediate disturbance (apart from increased citrate levels) or ATP increase (on the contrary, ATP gradually decreases throughout the 21 days, Figure 5g), perhaps due to its use in protein anabolism and rapid secretion to the extracellular region and the subsequent dephosphorylation for hydroxyapatite formation (Figure 6).^74−76^ The increase in citrate (%var._D14→D21_ = +49.2%) that follows the glycolysis increase (Figures 4 and 5e) may indeed suggest at least a partial TCA cycle enhanced activity mainly to form citrate (perhaps in tandem with glutaminolysis, as previously mentioned).^67^ Citrate has been extensively studied as a key player in bone metabolic regulation,^11,77^ having been proposed to bind to and stabilize hydroxyapatite crystals, preventing their further growth.^78^ Interestingly, however, citrate (and other TCA intermediates) has been seen to decrease by the end osteogenic differentiation of umbilical cord blood MSCs,^12^ in contrast with our results, which suggests that further studies are needed to fully understand the source and role of citrate variations in the osteogenic differentiation of different types of MSCs.

Creatine and its phosphorylated form (PCr) are also closely related to energy metabolism. Creatine kinase is responsible for the reversible phosphorylation of creatine by ATP, with PCr and creatine expected to vary conversely. However, opposite variations of the two metabolites are observed only between days 7 and 14 (Figure 6), which suggests that PCr (%var. = −21.8%) is in this stage being used to form creatine (%var. = +93.1%) and ATP, probably to help meet the high ATP demand of differentiating cells around day 7 of osteogenic differentiation (Figure 5d). Curiously, creatine kinase has been found in matrix vesicles isolated from chicken embryo femurs, seemingly contributing to the resynthesis of ATP at the expense of PCr.^79^ It is therefore possible that PCr may be released into matrix vesicles, thus justifying the gradual decrease in this compound until day 14.

### Cell Membrane Metabolism

Our results have shown a marked variation in the profiles of choline-containing compounds and ethanolamine (precursors of cell membrane phospholipids) throughout osteogenic differentiation (Figures 4 and 5f), revealing significantly active plasma membrane remodelling mechanisms. This is consistent with a previous MS-based lipidomic study reporting unique membrane features acquired during osteogenic and adipogenic differentiation, emphasizing the capacity for lineage-specific membrane remodelling.^13^ Our results indicate GPC accumulation (%var._D0→D21_ = +136.2%), a breakdown product of phosphatidylcholine (PtdCho), as a possible indicator of enhanced membrane degradation occurring as early as day 1 and continuing thereafter (Figure 5f). A choline increase until day 14 (%var._D0→D14_ = +383.0%, Figure 5f), without a concomitant GPC or PC decrease, may indicate a possible enhanced uptake of choline from extracellular media (not measured here), perhaps to promote membrane building for proliferating cells.^80^ Between days 7 and 14 (Figure 5f), intracellular enrichment in choline (%var. = +96.4%) and ethanolamine (%var. = +162.9%), along with a tendency for PC reduction (%var. = −29.2%), are consistent with an expected upregulation of the phosphoethanolamine/phosphocholine phosphatase (PHOSPHO1), which possesses a high affinity for PC and phosphoethanolamine (not directly detected here but for which ethanolamine is a precursor) in mineralizing cells, such as osteoblasts.^81^ PHOSPHO1 plays an important role in the initial steps of bone mineralization (Figure 6), generating Pi inside extracellular matrix vesicles released during osteogenic differentiation.^82^ In addition, the transporter-mediated influx of Pi produced by ALP (and other phosphatases or ATPases) promotes the formation of hydroxyapatite in matrix vesicles. Eventually, these undergo rupture, leading to hydroxyapatite deposition within the extracellular matrix, in the form of mineral nodules. After day 14 (Figure 5f), the decreases in choline (%var. = −37.2%) and ethanolamine (%var. = −31.5%) could contribute to the rise in PC levels (%var. = +44.8%). In addition, reduced choline levels near the end of osteogenic differentiation may also be related to the increasing tendency of betaine (clearly seen in two of the three samples from day 21), which is thought to enhance bone formation.^83^

### Nucleotides and Derivatives

The gradually lower ATP levels throughout osteogenic differentiation (%var._D0→D21_ = −57.3%, Figure 5g) and the consequent accumulation of ATP breakdown products after day 7 (ADP, AMP, and adenosine; %var._D7→14_ = +49.9 to +707.3%) reflect the expected high energy demand during the process, especially due to the extensive synthesis of extracellular matrix proteins. As previously mentioned, during osteogenic differentiation, ATP is most likely secreted to the extracellular media and increasingly dephosphorylated, with both processes contributing to the intracellular depletion observed here.^74,75,82,84^ Under specific conditions, extracellular ATP can, when hydrolyzed into adenosine by specialized ectoenzymes, potentiate osteogenic differentiation (Figure 6), increasing mineralization and RUNX2 expression.^85^ In the literature, altered levels of metabolites from nucleotide metabolism during osteogenic differentiation have been briefly mentioned by previous MS-based metabolomic studies.^11,17,18^

One of the main changes seen here regards the intracellular levels of UDP-GlcNAc (%var._D1→D7_ = +261.5%), a product of the hexosamine biosynthetic pathway, which can act as a substrate for enzymatic *O*-linked β-*N*-acetylglucosamine glycosylation, a dynamic post-translational modification of the Ser/Thr residues.^86^ Notably, there is evidence of *O*-glycosylation positively regulating transcription factors required for osteogenic differentiation, such as RUNX2,^87,88^ which peaks around day 7 and is downregulated in mature osteoblasts.^89^ The peaking levels of UDP-GlcNAc on day 7 may tentatively be interpreted as evidence of increased protein *O*-glycosylation processes in the intermediate stage of osteogenic differentiation.

NAD^+^ levels decreased until day 1 (%var. = −35.6%) and subsequently increased (Figure 5h), seemingly mirroring the trajectory of 1-MNA levels after day 7. Besides its role as an essential cofactor in several redox reactions (Figure 6), NAD^+^ can be cleaved into nicotinamide (NAM) by several enzymes that mediate the histone acetylation status (such as the deacetylase sirtuin (SIRT)), allowing for intimate crosstalk between the energetic metabolic status and the epigenome.^90^ Through the actions of nicotinamide phosphoribosyltransferase (NAMPT) and nicotinamide mononucleotide adenylyltransferase, NAM can be subsequentially converted into nicotinamide mononucleotide (NMN) and back to NAD^+^ through the NAD^+^ salvage pathway (Figure 6). Interestingly, NAMPT inhibition and consequent NAM accumulation have been previously associated with enhanced adipogenesis at the expense of osteogenic differentiation in MSC.^91^ In this study, the increasing tendency of NAD^+^ (%var._D7→D14_ = +79.4%, Figure 5h) in tandem with the decrease in 1-MNA (%var._D7→D14_ = −26.0%), with the latter resulting from the irreversible methylation of NAM by nicotinamide-*N*-methyltransferase (NNMT, Figure 6), may possibly be due to low levels of NAM, which, in turn, may promote osteogenic differentiation. A previous LC-MS-based metabolomic study reported an enrichment of extracellular 1-MNA throughout osteogenic differentiation, suggesting that metabolomic changes in NADH metabolism may reflect different stages of differentiation.^11^ The secretion of 1-MNA to the medium may explain the intracellular depletion of this metabolite observed here. We could also hypothesize that a possible decrease in NNMT activity/expression could contribute to decreased 1-MNA and allow NAD^+^ to be recycled. This could result in SIRT activation, most likely inducing osteogenic differentiation. Indeed, the NAD^+^ metabolism has been noted as a distinguishable feature between undifferentiated and differentiated cells, more particularly, for human MSCs (hMSCs) and human dermal fibroblasts.^92^ Whereas the NAD^+^/NADH redox ratio was stable during the replicative expansion of fibroblasts, undifferentiated hMSCs at later passages were associated with a marked accumulation of NADH, at the expense of NAD^+^, and a reduction of SIRT1 activity. In addition, SIRT6 has been described as an osteogenic promoter in AMSCs, enhancing mineralization and upregulating the expression of osteogenic-related genes.^93^

### GSH Metabolism

Antioxidative protection mechanisms during osteogenic differentiation have been already discussed in relation to particular amino acids (namely, phenylalanine and taurine), and the levels of GSH provide another clear indication that antioxidative mechanisms are in place in differentiating hAMSCs from day 1 (Figure 5a,c), keeping cells protected from ROS and other oxidative processes until day 21. GSH serves as an electron donor, acting as a nonenzymatic antioxidant, directly scavenging radical species, or generating the oxidized glutathione disulfide dimer.^94^ The balance between GSH and oxidized glutathione (GSSG) reflects the intracellular redox state and can be interpreted as an indicator of oxidative stress; however, GSSG could not be detected in our spectra. In any case, the GSH increase between days 7 and 14 (%var. = +28.2%, Figures 4 and 5c) indicates that antioxidative mechanisms are active during hAMSC osteogenic differentiation, in agreement with previous reports.^70,95^ The importance of GSH action in osteogenic differentiation has been further demonstrated through GSH and *N*-acetylcysteine (NAC, a GSH precursor) treatments of mouse calvarial cells, which resulted in the promotion of osteogenic differentiation.^96^ In another study, different GSH/GSSG ratios generated by GSH, NAC, or butionine sulfoximine (inhibitor of GSH synthesis) treatments influenced the expression of early and late osteogenic markers.^97^ Increasing evidence has suggested that the lineage commitment of MSCs is ROS-dependent, at least in part,^98^ and SIRT1 was also shown to be a key player in this process.^99^ Whereas an oxidized intracellular environment encourages the adipogenic differentiation of MSCs, excessive amounts of ROS seem to suppress osteogenic differentiation.^98^ In fact, intracellular ROS reportedly decrease during the course of osteogenesis, in parallel with the strong upregulation of antioxidant metabolites and enzymes, which is consistent with the rising levels of GSH observed here from day 1.^70^ Conversely, however, the enhanced osteogenic differentiation of human BMMSCs (hBMMSCs) has been associated with increased levels of ROS,^28^ although the concentration was suggested to be below the threshold that determines osteogenic suppression. Curiously, pyruvate has been suggested as a possible ROS scavenger,^100^ and an ROS-mediated pyruvate decarboxylation pathway could putatively justify the accumulation of acetate observed here (%var._D14→D21_ = +21.6%, Figure 6).

## Conclusions

Untargeted NMR metabolomics has been employed for the first time, to our knowledge, to characterize the intracellular metabolic adaptations of hAMSC during osteogenic differentiation. Meaningful changes in the levels of just over 30 metabolites are reported, indicating metabolic adaptations from days 0 to 1 and throughout the whole 21-day period. The earliest processes (days 0 to 1) include an enrichment tendency of many amino acid pools, although glutamate is taken up immediately (as well as glycine and aspartate, although to lower extents) probably due to early protein synthesis, supported by early ATP and PCr hydrolysis energetics (also leading to the enrichment of extracellular phosphate for hydroxyapatite synthesis). From day 1 to day 7, most amino acids are used for protein synthesis (as their levels steadily decrease) and possibly feed, at least in part, into the TCA cycle (although no significant changes in its intermediates are noted at this point), whereas ATP and PCr maintain their roles as sources of energy and inorganic phosphate. In addition to this, GSH begins to increase as a sign as antioxidative protection, GPC and choline increase, reflecting cell membrane remodelling, and a three-fold UDP-GlcNAc increase reflects an important adaptation of protein *O*-glycosylation reactions. Between days 7 and 14, strong glycolysis and gluminolytic enhancement occurs, possibly to produce citrate (increased after day 14) and to replenish glutamate levels for protein synthesis. During the same period, an interplay between NAD^+^, GSH, and ROS scavenging mechanisms seems to take place, along with strong activation of PCr dephosphorylation, and a distinct membrane remodelling pattern arises (with a rising trend for several membrane precursors increasing), probably related to the expected slowing down of cell proliferation rates. Between days 14 and 21, most processes tend to stabilize, although with protein synthesis, possible TCA cycle activity (and possibly OxPhos) enhancement, and membrane composition remodelling (with GPC accumulation and decreasing choline and ethanolamine) still ongoing. This work paves the way to characterizing the dynamic metabolism of hAMSCs during osteogenic differentiation, unveiling new metabolic biomarkers that will be potentially useful to monitor the efficacy of the osteogenic lineage commitment (which usually competes with adipogenic differentiation). In addition, the medium supplementation/depletion of key metabolites could affect the adipo-osteogenic balance and potentially improve the osteogenic differentiation.

Finally, it is important to recognize some limitations with this study. First, there is a limitation regarding the absence of a detailed profiling characterization of undifferentiated control cells at the time points under study, as cell aging in the same cell passage may contribute to some of the metabolite changes reported here. Second, limitations exist in relation to the low number of samples, which hinders a full robust statistical evaluation of stepwise metabolite changes, as well as to the absence of knowledge on how, and how significantly, the measured metabolite changes may differ between donors. Also, complementary studies on the accompanying lipidome and exometabolome changes will certainly complement the important knowledge on how hAMSCs adapt their metabolism during osteogenic differentiation, the subject of ongoing work in our group. Also, it would be of great interest to compare the results of the cell extracts shown here with the direct analysis of intact cells (or cell lysates) by high-resolution magic-angle spinning (HRMAS) NMR, which enables the simultaneous detection of both polar metabolites and lipids, although with relatively lower resolution.^101^ To our knowledge, this technique has been used only once in the context of MSC differentiation, more specifically, to monitor metabolic changes during adipogenic differentiation.^102^

## Supporting Information Available

Figure S1. Expression of osteogenic markers, namely, calcium and osteocalcin during hAMSC osteogenic differentiation. Figure S2. Partial least-squares discriminant analysis (PLS-DA) scores plot representing all sampling days of osteogenic differentiation of hAMSCs. Table S1. 500 MHz ^1^H NMR assignment of polar endometabolites identified in hAMSCs throughout osteogenic differentiation. Table S2. Main statistically significant metabolic differences during osteogenic differentiation of hAMSCs comparing extreme days 0 and 21 and classes before and after day 7 meeting the limiting criteria: |ES| > 0.50, ES error <80%, and Wilcoxon rank-sum test *p* value <0.05 (PDF)

## References

[ref1] Baht G. S.; Vi L.; Alman B. A. The Role of the Immune Cells in Fracture Healing. Curr. Osteoporos. Rep. 2018, 16 (2), 138–145. 10.1007/s11914-018-0423-2. 29508143 PMC5866272

[ref2] Gibon E.; Lu L.; Goodman S. B. Aging, Inflammation, Stem Cells, and Bone Healing. Stem Cell Res. Ther. 2016, 7 (1), Article No. 44 10.1186/s13287-016-0300-9. 27006071 PMC4804630

[ref3] Vidal L.; Kampleitner C.; Brennan M.; Hoornaert A.; Layrolle P. Reconstruction of Large Skeletal Defects: Current Clinical Therapeutic Strategies and Future Directions Using 3D Printing. Front. Bioeng. Biotechnol. 2020, 8, 61 10.3389/fbioe.2020.00061. 32117940 PMC7029716

[ref4] Younger E. M.; Chapman M. W. Morbidity at Bone Graft Donor Sites. J. Orthop. Trauma 1989, 3 (3), 192–195. 10.1097/00005131-198909000-00002. 2809818

[ref5] Kim D. H.; Rhim R.; Li L.; Martha J.; Swaim B. H.; Banco R. J.; Jenis L. G.; Tromanhauser S. G. Prospective Study of Iliac Crest Bone Graft Harvest Site Pain and Morbidity. Spine J. 2009, 9 (11), 886–892. 10.1016/j.spinee.2009.05.006. 19540168

[ref6] Iaquinta M. R.; Mazzoni E.; Bononi I.; Rotondo J. C.; Mazziotta C.; Montesi M.; Sprio S.; Tampieri A.; Tognon M.; Martini F. Adult Stem Cells for Bone Regeneration and Repair. Front. Cell Dev. Biol. 2019, 7, 268 10.3389/fcell.2019.00268. 31799249 PMC6863062

[ref7] Segers K.; Declerck S.; Mangelings D.; Heyden Y. V.; Eeckhaut A. V. Analytical Techniques for Metabolomic Studies: A Review. Bioanalysis 2019, 11 (24), 2297–2318. 10.4155/bio-2019-0014. 31845604

[ref8] Saccenti E.; Hoefsloot H. C. J.; Smilde A. K.; Westerhuis J. A.; Hendriks M. M. W. B. Reflections on Univariate and Multivariate Analysis of Metabolomics Data. Metabolomics 2014, 10 (3), 361–374. 10.1007/s11306-013-0598-6.

[ref9] Loeffler J.; Duda G. N.; Sass F. A.; Dienelt A. The Metabolic Microenvironment Steers Bone Tissue Regeneration. Trends Endocrinol. Metab. 2018, 29 (2), 99–110. 10.1016/j.tem.2017.11.008. 29290501

[ref10] Bispo D. S. C.; Jesus C. S. H.; Marques I. M. C.; Romek K. M.; Oliveira M. B.; Mano J. F.; Gil A. M. Metabolomic Applications in Stem Cell Research: A Review. Stem Cell Rev. Reports 2021, 17 (6), 2003–2024. 10.1007/s12015-021-10193-z. 34131883

[ref11] Surrati A.; Linforth R.; Fisk I. D.; Sottile V.; Kim D. H. Non-Destructive Characterisation of Mesenchymal Stem Cell Differentiation Using LC-MS-Based Metabolite Footprinting. Analyst 2016, 141 (12), 3776–3787. 10.1039/C6AN00170J. 27102615

[ref12] Klontzas M. E.; Vernardis S. I.; Heliotis M.; Tsiridis E.; Mantalaris A. Metabolomics Analysis of the Osteogenic Differentiation of Umbilical Cord Blood Mesenchymal Stem Cells Reveals Differential Sensitivity to Osteogenic Agents. Stem Cells Dev. 2017, 26 (10), 723–733. 10.1089/scd.2016.0315. 28418785 PMC5439454

[ref13] Levental K. R.; Surma M. A.; Skinkle A. D.; Lorent J. H.; Zhou Y.; Klose C.; Chang J. T.; Hancock J. F.; Levental I. W-3 Polyunsaturated Fatty Acids Direct Differentiation of the Membrane Phenotype in Mesenchymal Stem Cells To Potentiate Osteogenesis. Sci. Adv. 2017, 3 (11), eaao1193 10.1126/sciadv.aao1193. 29134198 PMC5677358

[ref14] Zhao G.; Zhong H.; Rao T.; Pan Z. Metabolomic Analysis Reveals That the Mechanism of Astaxanthin Improves the Osteogenic Differentiation Potential in Bone Marrow Mesenchymal Stem Cells. Oxid. Med. Cell. Longev. 2020, 2020, 3427430 10.1155/2020/3427430. 32308800 PMC7132583

[ref15] Hodgkinson T.; Tsimbouri P. M.; Llopis-Hernandez V.; Campsie P.; Scurr D.; Childs P. G.; Phillips D.; Donnelly S.; Wells J. A.; O’Brien F. J.; Salmeron-Sanchez M.; Burgess K.; Alexander M.; Vassalli M.; Oreffo R. O. C.; Reid S.; France D. J.; Dalby M. J. The Use of Nanovibration to Discover Specific and Potent Bioactive Metabolites That Stimulate Osteogenic Differentiation in Mesenchymal Stem Cells. Sci. Adv. 2021, 7 (9), eabb7921 10.1126/sciadv.abb7921. 33637520 PMC7909882

[ref16] Surrati A.; Evseev S.; Jourdan F.; Kim D.-H.; Sottile V. Osteogenic Response of Human Mesenchymal Stem Cells Analysed Using Combined Intracellular and Extracellular Metabolomic Monitoring. Cell. Physiol. Biochem. 2021, 55 (3), 311–326. 10.33594/000000377. 34148309

[ref17] Tsimbouri P. M.; McMurray R. J.; Burgess K. V.; Alakpa E. V.; Reynolds P. M.; Murawski K.; Kingham E.; Oreffo R. O. C.; Gadegaard N.; Dalby M. J. Using Nanotopography and Metabolomics to Identify Biochemical Effectors of Multipotency. ACS Nano 2012, 6 (11), 10239–10249. 10.1021/nn304046m. 23072705

[ref18] Seras-Franzoso J.; Tsimbouri P. M.; Burgess K. V.; Unzueta U.; Garcia-Fruitos E.; Vazquez E.; Villaverde A.; Dalby M. J. Topographically Targeted Osteogenesis of Mesenchymal Stem Cells Stimulated by Inclusion Bodies Attached to Polycaprolactone Surfaces. Nanomedicine 2014, 9 (2), 207–220. 10.2217/nnm.13.43. 23631503

[ref19] Tsimbouri P. M.; Childs P. G.; Pemberton G. D.; Yang J.; Jayawarna V.; Orapiriyakul W.; Burgess K.; González-García C.; Blackburn G.; Thomas D.; Vallejo-Giraldo C.; Biggs M. J. P.; Curtis A. S. G.; Salmerón-Sánchez M.; Reid S.; Dalby M. J. Stimulation of 3D Osteogenesis by Mesenchymal Stem Cells Using a Nanovibrational Bioreactor. Nat. Biomed. Eng. 2017, 1 (9), 758–770. 10.1038/s41551-017-0127-4. 31015671

[ref20] McNamara L. E.; Sjöström T.; Burgess K. E. V.; Kim J. J. W.; Liu E.; Gordonov S.; Moghe P. V.; Meek R. M. D.; Oreffo R. O. C.; Su B.; Dalby M. J. Skeletal Stem Cell Physiology on Functionally Distinct Titania Nanotopographies. Biomaterials 2011, 32 (30), 7403–7410. 10.1016/j.biomaterials.2011.06.063. 21820172

[ref21] Amer M. H.; Alvarez-Paino M.; McLaren J.; Pappalardo F.; Trujillo S.; Wong J. Q.; Shrestha S.; Abdelrazig S.; Stevens L. A.; Lee J. B.; Kim D.-H.; González-García C.; Needham D.; Salmerón-Sánchez M.; Shakesheff K. M.; Alexander M. R.; Alexander C.; Rose F. R. Designing Topographically Textured Microparticles for Induction and Modulation of Osteogenesis in Mesenchymal Stem Cell Engineering. Biomaterials 2021, 266 (9), 120450 10.1016/j.biomaterials.2020.120450. 33096376

[ref22] Yang J.; McNamara L. E.; Gadegaard N.; Alakpa E. V.; Burgess K. V.; Meek R. M. D.; Dalby M. J. Nanotopographical Induction of Osteogenesis through Adhesion, Bone Morphogenic Protein Cosignaling, and Regulation of MicroRNAs. ACS Nano 2014, 8 (10), 9941–9953. 10.1021/nn504767g. 25227207

[ref23] Klontzas M. E.; Reakasame S.; Silva R.; Morais J. C. F.; Vernardis S.; MacFarlane R. J.; Heliotis M.; Tsiridis E.; Panoskaltsis N.; Boccaccini A. R.; Mantalaris A. Oxidized Alginate Hydrogels with the GHK Peptide Enhance Cord Blood Mesenchymal Stem Cell Osteogenesis: A Paradigm for Metabolomics-Based Evaluation of Biomaterial Design. Acta Biomater. 2019, 88, 224–240. 10.1016/j.actbio.2019.02.017. 30772514

[ref24] Bow A.; Jackson B.; Griffin C.; Howard S.; Castro H.; Campagna S.; Biris A. S.; Anderson D. E.; Bourdo S.; Dhar M. Multiomics Evaluation of Human Fat-Derived Mesenchymal Stem Cells on an Osteobiologic Nanocomposite. Biores. Open Access 2020, 9 (1), 37–50. 10.1089/biores.2020.0005. 32117598 PMC7047255

[ref25] Mohamed-Ahmed S.; Fristad I.; Lie S. A.; Suliman S.; Mustafa K.; Vindenes H.; Idris S. B. Adipose-Derived and Bone Marrow Mesenchymal Stem Cells: A Donor-Matched Comparison. Stem Cell Res. Ther. 2018, 9, 168 10.1186/s13287-018-0914-1. 29921311 PMC6008936

[ref26] Mattar P.; Bieback K. Comparing the Immunomodulatory Properties of Bone Marrow, Adipose Tissue, and Birth-Associated Tissue Mesenchymal Stromal Cells. Front. Immunol. 2015, 6, 560 10.3389/fimmu.2015.00560. 26579133 PMC4630659

[ref27] Roberts J. N.; Sahoo J. K.; McNamara L. E.; Burgess K. V.; Yang J.; Alakpa E. V.; Anderson H. J.; Hay J.; Turner L. A.; Yarwood S. J.; Zelzer M.; Oreffo R. O. C.; Ulijn R. V.; Dalby M. J. Dynamic Surfaces for the Study of Mesenchymal Stem Cell Growth through Adhesion Regulation. ACS Nano 2016, 10 (7), 6667–6679. 10.1021/acsnano.6b01765. 27322014 PMC4963921

[ref28] Orapiriyakul W.; Tsimbouri M. P.; Childs P.; Campsie P.; Wells J.; Fernandez-Yague M. A.; Burgess K.; Tanner K. E.; Tassieri M.; Meek D.; Vassalli M.; Biggs M. J. P.; Salmeron-Sanchez M.; Oreffo R. O. C.; Reid S.; Dalby M. J. Nanovibrational Stimulation of Mesenchymal Stem Cells Induces Therapeutic Reactive Oxygen Species and Inflammation for Three-Dimensional Bone Tissue Engineering. ACS Nano 2020, 14 (8), 10027–10044. 10.1021/acsnano.0c03130. 32658450 PMC7458485

[ref29] McMurray R. J.; Gadegaard N.; Tsimbouri P. M.; Burgess K. V.; McNamara L. E.; Tare R.; Murawski K.; Kingham E.; Oreffo R. O. C.; Dalby M. J. Nanoscale Surfaces for the Long-Term Maintenance of Mesenchymal Stem Cell Phenotype and Multipotency. Nat. Mater. 2011, 10 (8), 637–644. 10.1038/nmat3058. 21765399

[ref30] Lorthongpanich C.; Thumanu K.; Tangkiettrakul K.; Jiamvoraphong N.; Laowtammathron C.; Damkham N.; U-Pratya Y.; Issaragrisil S. YAP as a Key Regulator of Adipo-Osteogenic Differentiation in Human MSCs. Stem Cell Res. Ther. 2019, 10 (1), 402 10.1186/s13287-019-1494-4. 31852542 PMC6921580

[ref31] Szpalski C.; Barbaro M.; Sagebin F.; Warren S. M. Bone Tissue Engineering: Current Strategies and Techniques-Part II: Cell Types. Tissue Eng. - Part B Rev. 2012, 18 (4), 258–269. 10.1089/ten.teb.2011.0440. 22224439

[ref32] Silva C. G. da; Barretto L. S. de S.; Lo Turco E. G.; Santos A. de L.; Lessio C.; Martins Júnior H. A.; Almeida F. G. de. Lipidomics of Mesenchymal Stem Cell Differentiation. Chem. Phys. Lipids 2020, 232, 104964 10.1016/j.chemphyslip.2020.104964. 32882223

[ref33] Alakpa E. V.; Jayawarna V.; Lampel A.; Burgess K. V.; West C. C.; Bakker S. C. J.; Roy S.; Javid N.; Fleming S.; Lamprou D. A.; Yang J.; Miller A.; Urquhart A. J.; Frederix P. W. J. M.; Hunt N. T.; Péault B.; Ulijn R. V.; Dalby M. J. Tunable Supramolecular Hydrogels for Selection of Lineage-Guiding Metabolites in Stem Cell Cultures. Chem. 2016, 1 (2), 298–319. 10.1016/j.chempr.2016.07.001.

[ref34] Klemenz; Meyer; Ekat; Bartels; Traxler; Schubert; Kamp; Miekisch; Peters Differences in the Emission of Volatile Organic Compounds (VOCs) between Non-Differentiating and Adipogenically Differentiating Mesenchymal Stromal/Stem Cells from Human Adipose Tissue. Cells 2019, 8 (7), 697 10.3390/cells8070697. 31295931 PMC6678290

[ref35] Rampler E.; Egger D.; Schoeny H.; Rusz M.; Pacheco M. P.; Marino G.; Kasper C.; Naegele T.; Koellensperger G. The Power of LC-MS Based Multiomics: Exploring Adipogenic Differentiation of Human Mesenchymal Stem/Stromal Cells. Molecules 2019, 24 (19), 3615 10.3390/molecules24193615. 31597247 PMC6804244

[ref36] Mitchell A.; Ashton L.; Yang X. B.; Goodacre R.; Smith A.; Kirkham J. Detection of Early Stage Changes Associated with Adipogenesis Using Raman Spectroscopy under Aseptic Conditions. Cytom. Part A 2015, 87 (11), 1012–1019. 10.1002/cyto.a.22777. PMC4832334 26441162

[ref37] Bhinderwala F.; Wase N.; DiRusso C.; Powers R. Combining Mass Spectrometry and NMR Improves Metabolite Detection and Annotation. J. Proteome Res. 2018, 17 (11), 4017–4022. 10.1021/acs.jproteome.8b00567. 30303385 PMC6668615

[ref38] Castiglione F.; Ferro M.; Mavroudakis E.; Pellitteri R.; Bossolasco P.; Zaccheo D.; Morbidelli M.; Silani V.; Mele A.; Moscatelli D.; Cova L. NMR Metabolomics for Stem Cell Type Discrimination. Sci. Rep. 2017, 7 (1), 15808 10.1038/s41598-017-16043-8. 29150616 PMC5693937

[ref39] Wu H.; Southam A. D.; Hines A.; Viant M. R. High-Throughput Tissue Extraction Protocol for NMR- and MS-Based Metabolomics. Anal. Biochem. 2008, 372 (2), 204–212. 10.1016/j.ab.2007.10.002. 17963684

[ref40] Wishart D. S.; Tzur D.; Knox C.; Eisner R.; Guo A. C.; Young N.; Cheng D.; Jewell K.; Arndt D.; Sawhney S.; Fung C.; Nikolai L.; Lewis M.; Coutouly M. A.; Forsythe I.; Tang P.; Shrivastava S.; Jeroncic K.; Stothard P.; Amegbey G.; Block D.; Hau D. D.; Wagner J.; Miniaci J.; Clements M.; Gebremedhin M.; Guo N.; Zhang Y.; Duggan G. E.; MacInnis G. D.; Weljie A. M.; Dowlatabadi R.; Bamforth F.; Clive D.; Greiner R.; Li L.; Marrie T.; Sykes B. D.; Vogel H. J.; Querengesser L. HMDB: The Human Metabolome Database. Nucleic Acids Res. 2007, 35 (1), D521–D526. 10.1093/nar/gkl923. 17202168 PMC1899095

[ref41] Veselkov K. A.; Lindon J. C.; Ebbels T. M. D.; Crockford D.; Volynkin V. V.; Holmes E.; Davies D. B.; Nicholson J. K. Recursive Segment-Wise Peak Alignment of Biological 1 H NMR Spectra for Improved Metabolic Biomarker Recovery. Anal. Chem. 2009, 81 (1), 56–66. 10.1021/ac8011544. 19049366

[ref42] Trygg J.; Holmes E.; Lundstedt T. Chemometrics in Metabonomics. J. Proteome Res. 2007, 6 (2), 469–479. 10.1021/pr060594q. 17269704

[ref43] Bridge P. D.; Sawilowsky S. S. Increasing Physicians’ Awareness of the Impact of Statistics on Research Outcomes: Comparative Power of the t-Test and Wilcoxon Rank-Sum Test in Small Samples Applied Research. J. Clin. Epidemiol. 1999, 52 (3), 229–235. 10.1016/S0895-4356(98)00168-1. 10210240

[ref44] Berben L.; Sereika S. M.; Engberg S. Effect Size Estimation: Methods and Examples. Int. J. Nurs. Stud. 2012, 49 (8), 1039–1047. 10.1016/j.ijnurstu.2012.01.015. 22377339

[ref45] Ranstam J. Multiple P-Values and Bonferroni Correction. Osteoarthritis and Cartilage 2016, 24 (May), 763–764. 10.1016/j.joca.2016.01.008. 26802548

[ref46] Cloarec O.; Dumas M.-E.; Craig A.; Barton R. H.; Trygg J.; Hudson J.; Blancher C.; Gauguier D.; Lindon J. C.; Holmes E.; Nicholson J. Statistical Total Correlation Spectroscopy: An Exploratory Approach for Latent Biomarker Identification from Metabolic 1 H NMR Data Sets. Anal. Chem. 2005, 77 (5), 1282–1289. 10.1021/ac048630x. 15732908

[ref47] Golub E. E. Role of Matrix Vesicles in Biomineralization. Biochim. Biophys. Acta - Gen. Subj. 2009, 1790 (12), 1592–1598. 10.1016/j.bbagen.2009.09.006. PMC2783689 19786074

[ref48] Stein G. S.; Lian J. B. Molecular Mechanisms Mediating Proliferation/Differentiation Interrelationships During Progressive Development of the Osteoblast Phenotype. Endocr. Rev. 1993, 14 (4), 424–442. 10.1210/edrv-14-4-424. 8223340

[ref49] Lefevre C.; Panthu B.; Naville D.; Guibert S.; Pinteur C.; Elena-Herrmann B.; Vidal H.; Rautureau G. J. P.; Mey A. Metabolic Phenotyping of Adipose-Derived Stem Cells Reveals a Unique Signature and Intrinsic Differences between Fat Pads. Stem Cells Int. 2019, 2019, 9323864 10.1155/2019/9323864. 31223312 PMC6541987

[ref50] Lee S. J.; Yi T. G.; Ahn S. H.; Lim D. K.; Kim S. na; Lee H. J.; Cho Y. K.; Lim J. Y.; Sung J. H.; Yun J. H.; Lim J.; Song S. U.; Kwon S. W. Comparative Study on Metabolite Level in Tissue-Specific Human Mesenchymal Stem Cells by an Ultra-Performance Liquid Chromatography Quadrupole Time of Flight Mass Spectrometry. Anal. Chim. Acta 2018, 1024, 112–122. 10.1016/j.aca.2018.04.018. 29776537

[ref51] Li J. Z.; Qu H.; Wu J.; Zhang F.; Jia Z. B.; Sun J. Y.; Lv B.; Kang Y.; Jiang S. L.; Kang K. Metabolic Profiles of Adipose-Derived and Bone Marrow-Derived Stromal Cells from Elderly Coronary Heart Disease Patients by Capillary Liquid Chromatography Quadrupole Time-of-Flight Mass Spectrometry. Int. J. Mol. Med. 2017, 41 (1), 184–194. 10.3892/ijmm.2017.3198. 29115374 PMC5746296

[ref52] Mastrangelo A.; Panadero M. I.; Perez L. M.; Galvez B. G.; Garcia A.; Barbas C.; Ruperez F. J. New Insight on Obesity and Adipose-Derived Stem Cells Using Comprehensive Metabolomics. Biochem. J. 2016, 473 (14), 2187–2203. 10.1042/BCJ20160241. 27208167

[ref53] Peng L.; Jia Z.; Yin X.; Zhang X.; Liu Y.; Chen P.; Ma K.; Zhou C. Comparative Analysis of Mesenchymal Stem Cells from Bone Marrow, Cartilage, and Adipose Tissue. Stem Cells Dev. 2008, 17 (4), 761–773. 10.1089/scd.2007.0217. 18393634

[ref54] Beck J.; Zerler B.; Moran E. Gene Array Analysis of Osteoblast Differentiation. Cell Growth Differ. 2001, 12 (2), 61–83. 11243467

[ref55] Paiva K. B. S.; Granjeiro J. M. Matrix Metalloproteinases in Bone Resorption, Remodeling, and Repair. Progress in Molecular Biology and Translational Science 2017, 148, 203–303. 10.1016/bs.pmbts.2017.05.001. 28662823

[ref56] Li J.; Wang Z.; Huang X.; Wang Z.; Chen Z.; Wang R.; Chen Z.; Liu W.; Wu B.; Fang F.; Qiu W. Dynamic Proteomic Profiling of Human Periodontal Ligament Stem Cells during Osteogenic Differentiation. Stem Cell Res. Ther. 2021, 12, 98 10.1186/s13287-020-02123-6. 33536073 PMC7860046

[ref57] Komori T. What Is the Function of Osteocalcin?. J. Oral Biosci. 2020, 62 (3), 223–227. 10.1016/j.job.2020.05.004. 32535287

[ref58] Butler W. T. The Nature and Significance of Osteopontin. Connect. Tissue Res. 1989, 23 (2–3), 123–136. 10.3109/03008208909002412. 2698313

[ref59] Li P.; Wu G. Roles of Dietary Glycine, Proline, and Hydroxyproline in Collagen Synthesis and Animal Growth. Amino Acids 2018, 50, 29–38. 10.1007/s00726-017-2490-6. 28929384

[ref60] Kim H.-J.; Kim W.-J.; Ryoo H.-M. Post-Translational Regulations of Transcriptional Activity of RUNX2. Mol. Cells 2020, 43 (2), 160–167. 10.14348/molcells.2019.0247. 31878768 PMC7057842

[ref61] Otto F.; Lübbert M.; Stock M. Upstream and Downstream Targets of RUNX Proteins. J. Cell. Biochem. 2003, 89 (1), 9–18. 10.1002/jcb.10491. 12682904

[ref62] Moriishi T.; Ozasa R.; Ishimoto T.; Nakano T.; Hasegawa T.; Miyazaki T.; Liu W.; Fukuyama R.; Wang Y.; Komori H.; Qin X.; Amizuka N.; Komori T. Osteocalcin Is Necessary for the Alignment of Apatite Crystallites, but Not Glucose Metabolism, Testosterone Synthesis, or Muscle Mass. PLOS Genet. 2020, 16 (5), e1008586 10.1371/journal.pgen.1008586. 32463816 PMC7255595

[ref63] Asou Y.; Rittling S. R.; Yoshitake H.; Tsuji K.; Shinomiya K.; Nifuji A.; Denhardt D. T.; Noda M. Osteopontin Facilitates Angiogenesis, Accumulation of Osteoclasts, and Resorption in Ectopic Bone. Endocrinology 2001, 142 (3), 1325–1332. 10.1210/endo.142.3.8006. 11181551

[ref64] Addison W. N.; Azari F.; Sørensen E. S.; Kaartinen M. T.; McKee M. D. Pyrophosphate Inhibits Mineralization of Osteoblast Cultures by Binding to Mineral, up-Regulating Osteopontin, and Inhibiting Alkaline Phosphatase Activity. J. Biol. Chem. 2007, 282 (21), 15872–15883. 10.1074/jbc.M701116200. 17383965

[ref65] Karner C. M.; Esen E.; Okunade A. L.; Patterson B. W.; Long F. Increased Glutamine Catabolism Mediates Bone Anabolism in Response to WNT Signaling. J. Clin. Invest. 2015, 125 (2), 551–562. 10.1172/JCI78470. 25562323 PMC4319407

[ref66] Yu Y.; Newman H.; Shen L.; Sharma D.; Hu G.; Mirando A. J.; Zhang H.; Knudsen E.; Zhang G.-F.; Hilton M. J.; Karner C. M. Glutamine Metabolism Regulates Proliferation and Lineage Allocation in Skeletal Stem Cells. Cell Metab. 2019, 29 (4), 966–978. 10.1016/j.cmet.2019.01.016. 30773468 PMC7062112

[ref67] Fu X.; Li Y.; Huang T.; Yu Z.; Ma K.; Yang M.; Liu Q.; Pan H.; Wang H.; Wang J.; Guan M. Runx2/Osterix and Zinc Uptake Synergize to Orchestrate Osteogenic Differentiation and Citrate Containing Bone Apatite Formation. Adv. Sci. 2018, 5 (4), 1700755 10.1002/advs.201700755. PMC5908346 29721422

[ref68] Lou J.; Han D.; Yu H.; Yu G.; Jin M.; Kim S. J. Cytoprotective Effect of Taurine against Hydrogen Peroxide-Induced Oxidative Stress in Umr-106 Cells through the Wnt/β-Catenin Signaling Pathway. Biomol. Ther. 2018, 26 (6), 584–590. 10.4062/biomolther.2018.049. PMC6254645 30060293

[ref69] Zhou C.; Zhang X.; Xu L.; Wu T.; Cui L.; Xu D. Taurine Promotes Human Mesenchymal Stem Cells to Differentiate into Osteoblast through the ERK Pathway. Amino Acids 2014, 46 (7), 1673–1680. 10.1007/s00726-014-1729-8. 24677149

[ref70] Chen C.-T.; Shih Y.-R. V.; Kuo T. K.; Lee O. K.; Wei Y.-H. Coordinated Changes of Mitochondrial Biogenesis and Antioxidant Enzymes During Osteogenic Differentiation of Human Mesenchymal Stem Cells. Stem Cells 2008, 26 (4), 960–968. 10.1634/stemcells.2007-0509. 18218821

[ref71] Pattappa G.; Heywood H. K.; de Bruijn J. D.; Lee D. A. The Metabolism of Human Mesenchymal Stem Cells during Proliferation and Differentiation. J. Cell. Physiol. 2011, 226 (10), 2562–2570. 10.1002/jcp.22605. 21792913

[ref72] Shares B. H.; Busch M.; White N.; Shum L.; Eliseev R. A. Active Mitochondria Support Osteogenic Differentiation by Stimulating-Catenin Acetylation. J. Biol. Chem. 2018, 293 (41), 16019–16027. 10.1074/jbc.RA118.004102. 30150300 PMC6187642

[ref73] Shum L. C.; White N. S.; Mills B. N.; De Mesy Bentley K. L.; Eliseev R. A. Energy Metabolism in Mesenchymal Stem Cells during Osteogenic Differentiation. Stem Cells Dev. 2016, 25 (2), 114–122. 10.1089/scd.2015.0193. 26487485 PMC4733323

[ref74] Roszek K.; Wujak M. How to Influence the Mesenchymal Stem Cells Fate? Emerging Role of Ectoenzymes Metabolizing Nucleotides. J. Cell. Physiol. 2019, 234 (1), 320–334. 10.1002/jcp.26904. 30078187

[ref75] Ciancaglini P.; Yadav M. C.; Sper Simão A. M.; Narisawa S.; Pizauro J. M.; Farquharson C.; Hoylaerts M. F.; Millán J. L. Kinetic Analysis of Substrate Utilization by Native and TNAP-, NPP1-, or PHOSPHO1-Deficient Matrix Vesicles. J. Bone Miner. Res. 2009, 25 (4), 716–723. 10.1359/jbmr.091023. PMC3153326 19874193

[ref76] Ansari S.; de Wildt B. W. M.; Vis M. A. M.; de Korte C. E.; Ito K.; Hofmann S.; Yuana Y. Matrix Vesicles: Role in Bone Mineralization and Potential Use as Therapeutics. Pharmaceuticals 2021, 14 (4), 289 10.3390/ph14040289. 33805145 PMC8064082

[ref77] Granchi D.; Baldini N.; Ulivieri F. M.; Caudarella R. Role of Citrate in Pathophysiology and Medical Management of Bone Diseases. Nutrients 2019, 11 (11), 2576 10.3390/nu11112576. 31731473 PMC6893553

[ref78] Hu Y. Y.; Rawal A.; Schmidt-Rohr K. Strongly Bound Citrate Stabilizes the Apatite Nanocrystals in Bone. Proc. Natl. Acad. Sci. U. S. A. 2010, 107 (52), 22425–22429. 10.1073/pnas.1009219107. 21127269 PMC3012505

[ref79] Sekrecka-Belniak A.; Balcerzak M.; Buchet R.; Pikula S. Active Creatine Kinase Is Present in Matrix Vesicles Isolated from Femurs of Chicken Embryo: Implications for Bone Mineralization. Biochem. Biophys. Res. Commun. 2010, 391 (3), 1432–1436. 10.1016/j.bbrc.2009.12.083. 20026305

[ref80] Roci I.; Watrous J. D.; Lagerborg K. A.; Jain M.; Nilsson R. Mapping Choline Metabolites in Normal and Transformed Cells. Metabolomics 2020, 16 (12), 125 10.1007/s11306-020-01749-0. 33249526 PMC7701132

[ref81] Roberts S. J.; Stewart A. J.; Sadler P. J.; Farquharson C. Human PHOSPHO1 Exhibits High Specific Phosphoethanolamine and Phosphocholine Phosphatase Activities. Biochem. J. 2004, 382 (1), 59–65. 10.1042/BJ20040511. 15175005 PMC1133915

[ref82] Yadav M. C.; Simão A. M. S.; Narisawa S.; Huesa C.; McKee M. D.; Farquharson C.; Millán J. L. Loss of Skeletal Mineralization by the Simultaneous Ablation of PHOSPHO1 and Alkaline Phosphatase Function: A Unified Model of the Mechanisms of Initiation of Skeletal Calcification. J. Bone Miner. Res. 2011, 26 (2), 286–297. 10.1002/jbmr.195. 20684022 PMC3179344

[ref83] Villa I.; Senesi P.; Montesano A.; Ferraretto A.; Vacante F.; Spinello A.; Bottani M.; Bolamperti S.; Rubinacci A.; Luzi L.; Terruzzi I. Betaine Promotes Cell Differentiation of Human Osteoblasts in Primary Culture. J. Transl. Med. 2017, 15 (1), 132 10.1186/s12967-017-1233-5. 28592272 PMC5463390

[ref84] Orriss I. R.; Knight G. E.; Utting J. C.; Taylor S. E. B.; Burnstock G.; Arnett T. R. Hypoxia Stimulates Vesicular ATP Release from Rat Osteoblasts. J. Cell. Physiol. 2009, 220 (1), 155–162. 10.1002/jcp.21745. 19259945

[ref85] Ciciarello M.; Zini R.; Rossi L.; Salvestrini V.; Ferrari D.; Manfredini R.; Lemoli R. M. Extracellular Purines Promote the Differentiation of Human Bone Marrow-Derived Mesenchymal Stem Cells to the Osteogenic and Adipogenic Lineages. Stem Cells Dev. 2013, 22 (7), 1097–1111. 10.1089/scd.2012.0432. 23259837 PMC3608030

[ref86] Santoyo-Ramos P.; Cristina M.; Robles-Flores M. The Role of O-Linked β-N-Acetylglucosamine (GlcNAc) Modification in Cell Signaling. In Glycosylation; InTech, 2012; pp 287–300.

[ref87] Nagel A. K.; Ball L. E. O-GlcNAc Modification of the Runt-Related Transcription Factor 2 (Runx2) Links Osteogenesis and Nutrient Metabolism in Bone Marrow Mesenchymal Stem Cells. Mol. Cell. Proteomics 2014, 13 (12), 3381–3395. 10.1074/mcp.M114.040691. 25187572 PMC4256491

[ref88] Koyama T.; Kamemura K. Global Increase in O-Linked N-Acetylglucosamine Modification Promotes Osteoblast Differentiation. Exp. Cell Res. 2015, 338 (2), 194–202. 10.1016/j.yexcr.2015.08.009. 26302267

[ref89] Komori T. Regulation of Proliferation, Differentiation and Functions of Osteoblasts by Runx2. Int. J. Mol. Sci. 2019, 20 (7), 1694 10.3390/ijms20071694. 30987410 PMC6480215

[ref90] Roberti A.; Fernández A. F.; Fraga M. F. Nicotinamide N-Methyltransferase: At the Crossroads between Cellular Metabolism and Epigenetic Regulation. Mol. Metab. 2021, 45 (5), 101165 10.1016/j.molmet.2021.101165. 33453420 PMC7868988

[ref91] Li Y.; He X.; Li Y.; He J.; Anderstam B.; Andersson G.; Lindgren U. Nicotinamide Phosphoribosyltransferase (Nampt) Affects the Lineage Fate Determination of Mesenchymal Stem Cells: A Possible Cause for Reduced Osteogenesis and Increased Adipogenesis in Older Individuals. J. Bone Miner. Res. 2011, 26 (11), 2656–2664. 10.1002/jbmr.480. 21812028

[ref92] Yuan X.; Liu Y.; Bijonowski B. M.; Tsai A.-C.; Fu Q.; Logan T. M.; Ma T.; Li Y. NAD+/NADH Redox Alterations Reconfigure Metabolism and Rejuvenate Senescent Human Mesenchymal Stem Cells in Vitro. Commun. Biol. 2020, 3 (1), 774 10.1038/s42003-020-01514-y. 33319867 PMC7738682

[ref93] Jia B.; Chen J.; Wang Q.; Sun X.; Han J.; Guastaldi F.; Xiang S.; Ye Q.; He Y. SIRT6 Promotes Osteogenic Differentiation of Adipose-Derived Mesenchymal Stem Cells Through Antagonizing DNMT1. Front. Cell Dev. Biol. 2021, 9, 648627 10.3389/fcell.2021.648627. 34239868 PMC8258422

[ref94] Kurutas E. B. The Importance of Antioxidants Which Play the Role in Cellular Response against Oxidative/Nitrosative Stress: Current State. Nutr. J. 2015, 15 (1), 71 10.1186/s12937-016-0186-5. PMC4960740 27456681

[ref95] Valle-Prieto A.; Conget P. A. Human Mesenchymal Stem Cells Efficiently Manage Oxidative Stress. Stem Cells Dev. 2010, 19 (12), 1885–1893. 10.1089/scd.2010.0093. 20380515

[ref96] Jun J. H.; Lee S.-H.; Kwak H. B.; Lee Z. H.; Seo S.-B.; Woo K. M.; Ryoo H.-M.; Kim G.-S.; Baek J.-H. N-Acetylcysteine Stimulates Osteoblastic Differentiation of Mouse Calvarial Cells. J. Cell. Biochem. 2008, 103 (4), 1246–1255. 10.1002/jcb.21508. 17979115

[ref97] Romagnoli C.; Marcucci G.; Favilli F.; Zonefrati R.; Mavilia C.; Galli G.; Tanini A.; Iantomasi T.; Brandi M. L.; Vincenzini M. T. Role of GSH/GSSG Redox Couple in Osteogenic Activity and Osteoclastogenic Markers of Human Osteoblast-like SaOS-2 Cells. FEBS J. 2012, 280 (3), 867–879. 10.1111/febs.12075. 23176170

[ref98] Atashi F.; Modarressi A.; Pepper M. S. The Role of Reactive Oxygen Species in Mesenchymal Stem Cell Adipogenic and Osteogenic Differentiation: A Review. Stem Cells Dev. 2015, 24 (10), 1150–1163. 10.1089/scd.2014.0484. 25603196 PMC4424969

[ref99] Lin C.-H.; Li N.-T.; Cheng H.-S.; Yen M.-L. Oxidative Stress Induces Imbalance of Adipogenic/Osteoblastic Lineage Commitment in Mesenchymal Stem Cells through Decreasing SIRT1 Functions. J. Cell. Mol. Med. 2017, 22 (2), 786–796. 10.1111/jcmm.13356. 28975701 PMC5783884

[ref100] Liu X.; Cooper D. E.; Cluntun A. A.; Warmoes M. O.; Zhao S.; Reid M. A.; Liu J.; Lund P. J.; Lopes M.; Garcia B. A.; Wellen K. E.; Kirsch D. G.; Locasale J. W. Acetate Production from Glucose and Coupling to Mitochondrial Metabolism in Mammals. Cell 2018, 175 (2), 502–513.e13. 10.1016/j.cell.2018.08.040. 30245009 PMC6173642

[ref101] Duarte I. F.; Marques J.; Ladeirinha A. F.; Rocha C.; Lamego I.; Calheiros R.; Silva T. M.; Marques M. P. M.; Melo J. B.; Carreira I. M.; Gil A. M. Analytical Approaches toward Successful Human Cell Metabolome Studies by NMR Spectroscopy. Anal. Chem. 2009, 81 (12), 5023–5032. 10.1021/ac900545q. 19462963

[ref102] Shi C.; Wang X.; Wu S.; Zhu Y.; Chung L. W. K.; Mao H. HRMAS 1 H-NMR Measured Changes of the Metabolite Profile as Mesenchymal Stem Cells Differentiate to Targeted Fat Cells in Vitro : Implications for Non-Invasive Monitoring of Stem Cell Differentiation in Vivo. J. Tissue Eng. Regen. Med. 2008, 2 (8), 482–490. 10.1002/term.120. 18932127 PMC4112179

