# Supplementary material for: NMR Metabolomics Assessment of Osteogenic Differentiation of Adipose-Tissue-Derived Mesenchymal Stem Cells
Source: J Proteome Res. 2022 Jan 21;21(3):654–70. doi: 10.1021/acs.jproteome.1c00832 (PMC9776527; doi:10.1021/acs.jproteome.1c00832)
Supplement: Supplementary file 1 — pr1c00832_si_001.pdf [file pr1c00832_si_001.pdf]

Supporting Information for

NMR metabolomics assessment of osteogenic differentiation of adipose tissue-derived mesenchymal stem cells

Daniela S. C. Bispo[1], Catarina S. H. Jesus[1], Marlene Correia[1], Filipa Ferreira[1], Giulia Bonifazio[1, 2], Brian J. Goodfellow[1], Mariana B. Oliveira[1], João F. Mano[1], Ana M. Gil[1,]*

[1] Department of Chemistry, CICECO - Aveiro Institute of Materials (CICECO/UA), University of Aveiro, Campus Universitario de Santiago, 3810-193 Aveiro, Portugal.

[2] University of Pavia, Department of Biotechnology Lazzaro Spallanzani, University of Pavia Corso Str. Nuova, 65, 27100 Pavia PV, Italy

Supporting Information includes:

Figure S1. Expression of osteogenic markers, namely calcium (Ca^{2+}) and osteocalcin (OCN) during hAMSCs osteogenesis. a) calcium quantification in cell lysates for days 7, 14 and 21 represented as Ca^{2+} total concentration present in culture flasks normalized by the total dsDNA; b) ELISA immunoassay quantification of OCN in media samples collected at days 14 and 21 represented as total concentration present in culture flasks, normalized by the total dsDNA; *: p value < 0.05.

Figure S2. Partial least-squares discriminant analysis (PLS-DA) scores plot representing all sampling days of osteogenic differentiation of hAMSCs. D_i: day i of osteogenic differentiation.

Table S1. 500 MHz ^{1}H NMR assignment of polar endo-metabolites identified in hAMSCs throughout osteogenic differentiation. AMP, adenosine monophosphate; ADP, Adenosine diphosphate; ATP, adenosine triphosphate; NAD^+, nicotinamide adenine

dinucleotide (oxidized); UDP-GalNAc, uridine diphospho-*N*-acetylgalactosamine; UDP-GlcNAc, uridine diphospho-*N*-acetylglucosamine. Multiplicity: s, singlet; d, doublet; dd, doublet of doublets; dt, doublet of triplets; t, triplet; q, quartet; m, multiplet. The second column indicates the metabolite ID numbers found in both the Human Metabolome Database (HMDB) and the Kyoto encyclopedia of genes and genomes (KEGG, https://www.genome.jp/kegg/).

Table S2. Main statistically significant metabolic differences during osteogenesis of hAMSCs comparing extreme days 0 and 21, and classes before and after day 7 meeting the limiting criteria: |ES| > 0.50, ES error < 80% and Wilcoxon Rank-sum test p-value < 0.05. Effect size (ES) was calculated according to reference 43 (positive and negative ES values correspond to metabolite level increases and decreases, respectively). [a]: peak used for integration (part of the spin system); [b]: note that *p*-values for comparisons of days 0 and 21 are not shown since they bear no statistical strength due to the low number of samples per group (although they remain < 0.05), yet all changes listed have been confirmed by visual inspection of the spectra; [c]: p-values that remain statistically significant upon Bonferroni correction,[44] where cut-off *p*-values of 4.44×10^{-3} (extreme days) and 1.92×10^{-3} (before and after day 7) were used. Abbreviations: GSH, glutathione (reduced); NAD^+, nicotinamide adenine dinucleotide (oxidized); other metabolites abbreviated as shown in Table 1. Resonance multiplicity: d, doublet; dd, doublet of doublets; m, multiplet; t, triplet; s, singlet; br, broad resonance. D_i: day i; U_δ: unassigned signal at chemical shift δ.

Figure S1

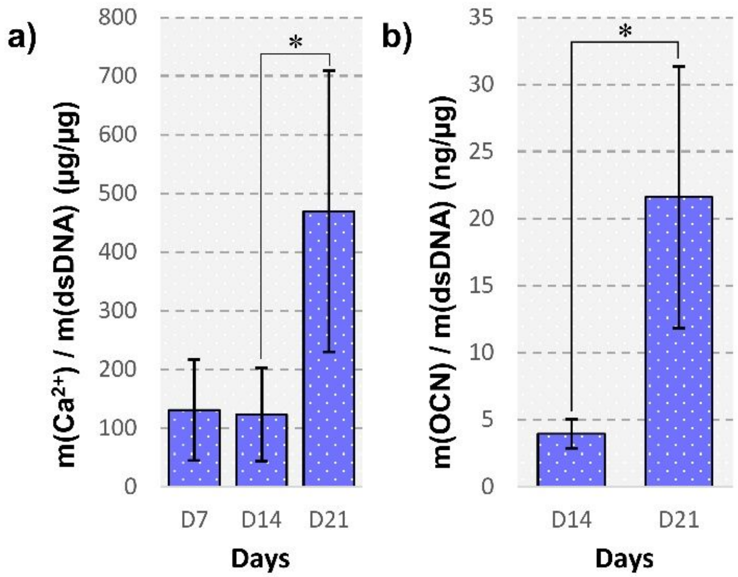

a)
800
700
600
500
400
300
200
100
0
*
m(Ca²⁺) / m(dsDNA) (µg/µg)
D7
D14
D21
Days
b)
35
30
25
20
15
10
5
0
*
m(OCN) / m(dsDNA) (ng/µg)
D14
D21
Days

Figure S2

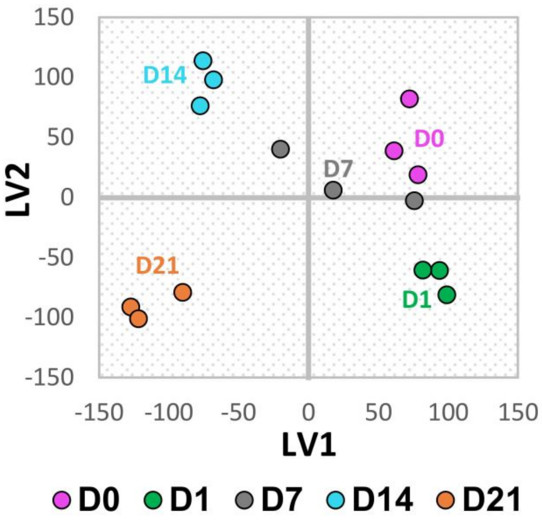
150
100
50
0
-50
-100
-150
LV2
-150
-100
-50
0
50
100
150
LV1
D14
D0
D7
D21
D1
D0
D1
D7
D14
D21

Table S1

Metabolite	HMDB ID, KEGG ID	δ ^{1}H in ppm (multiplicity, assignment)
1-Methylnicotinamide	HMDB0003152, C02918	4.49 (s, N-C**H**$_3$), 8.91 (d, 4-C**H**), 8.98 (d, 6-C**H**), 9.29 (s, 2-C**H**)
Acetate	HMDB0000042, C00033	1.92 (s, β-C**H**$_3$)
Acetone	HMDB0001659, C00207	2.24 (s, C**H**$_3$)
Adenosine	HMDB0000050, C00212	3.84/3.92 (dd/dd, 5'-CH ribose), 4.30 (q, 4'-CH ribose), 4.44 (dd, 3'-CH ribose), 6.08 (d, 1'-CH ribose), 8.27 (s, 2-CH ring), 8.35 (s, 8-CH ring)
ADP	HMDB0001341, C00008	4.24 (m, 5'-C**H**$_2$ ribose), 4.39 (m, 4'-C**H** ribose), 4.62 (m, 3'-C**H** ribose), 6.15 (d, 1'-C**H** ribose), 8.28 (s, 2-C**H** ring), 8.54 (s, 8-C**H** ring)
Alanine	HMDB0000161, C00041	1.48 (d, β-C**H**$_3$), 3.78 (q, α-C**H**)
AMP	HMDB0000045, C00020	4.02 (m, 5'-C**H**$_2$ ribose), 4.51 (m, 3'-C**H** ribose),6.15 (d, 1'-C**H** ribose), 8.28 (s, 2-C**H** ring), 8.62 (s, 8-C**H** ring)
Asparagine	HMDB0000168, C00152	2.86/2.95 (dd/dd, β-C**H**$_2$), 4.00 (dd, α-C**H**)
Aspartate	HMDB0000191, C00049	2.68/2.82 (dd/dd, β-C**H**$_2$), 3.90 (dd, α-C**H**)
ATP	HMDB0000538, C00002	4.22/4.29 (m/m, 5'-C**H**$_2$ ribose), 4.41 (m, 4'-C**H** ribose), 4.62 (m, 3'-C**H** ribose), 6.15 (d, 1'-C**H** ribose), 8.28 (s, 2-C**H** ring), 8.55 (s, 8-C**H** ring)
Betaine	HMDB0000043, C00719	3.27 (s, C**H**$_3$)
Choline	HMDB0000097, C00114	3.21 (s, N(C**H**$_3$)$_3$), 3.53 (m, N-C**H**$_2$), 4.07 (m, C**H**$_2$-OH)
Citrate	HMDB0000094, C00158	2.54 (d, α-C**H** & β-C**H**), 2.66 (d, α'-C**H** & β'-C**H**)
Creatine	HMDB0000064, C00300	3.04 (s, N-C**H**$_3$), 3.93 (s, N-C**H**$_2$)
Creatinine	HMDB0000562, C00791	3.05 (s, N-C**H**$_3$), 4.06 (s, N-C**H**$_2$)
Dimethylamine	HMDB0000087, C00543	2.73 (s, C**H**$_3$)
Ethanolamine	HMDB0000149, C00189	3.14 (t, C**H**$_2$-NH$_2$), 3.82 (t, C**H**$_2$-OH)
Formate	HMDB0000142, C00058	8.46 (s, HO-**H**C=O)
α-Glucose	HMDB0003345, C00031	5.24 (d, 1-C**H**)
β-Glucose	HMDB0000122, C00221	4.65 (d, 1-C**H**)
Glutamate	HMDB0000148, C00025	2.05/2.14 (m/m, β-C**H**$_2$), 2.35 (m, γ-C**H**$_2$), 3.76 (dd, α-C**H**)
Glutamine	HMDB0000641, C00064	2.14 (m, β-C**H**$_2$), 2.46 (m, γ-C**H**$_2$), 3.78 (t, α-C**H**)
Glutathione (reduced)	HMDB0000125, C00051	2.15 (m, β-C**H**$_2$ Glu), 2.55 (m, γ-C**H**$_2$ Glu), 2.96 (m, β-C**H**$_2$ Cys), 3.78 (m, α-C**H** Glu & α-C**H**$_2$, Gly), 4.57 (m, α-C**H** Cys)
Glycerophosphocholine	HMDB0000086, C00670	3.24 (s, N(C**H**$_3$)$_3$)
Glycine	HMDB0000123, C00037	3.56 (s, α-C**H**$_2$)
Hippurate	HMDB0000714, C01586	7.55 (t, 2-C**H** ring & 6-C**H** ring), 7.64 (t, 3-C**H** ring & 5-C**H** ring), 7.84 (d, 4- C**H** ring)
Histidine	HMDB0000177, C00135	7.08 (s, 5-C**H** ring), 7.84 (s, 2-C**H**)
Isoleucine	HMDB0000172, C00407	0.94 (t, δ-C**H**$_3$), 1.02 (d, γ'-C**H**$_3$), 1.28/1.45 (m/m, γ-C**H**$_2$), 1.98 (m, β-C**H**), 3.66 (d, α-C**H**)
Lactate	HMDB0000190, C00186	1.33 (d, C**H**$_3$), 4.11 (q, C**H**)
Leucine	HMDB0000687, C00123	0.96 (d, δ'-C**H**$_3$), 0.97 (d, δ-C**H**$_3$), 1.72 (m, γ-C**H** & β-C**H**$_2$), 3.75 (m, α-C**H**)
Lysine	HMDB0000182, C00047	1.48 (m, γ-C**H**$_2$), 1.72 (m, δ-C**H**$_2$), 1.91 (m, β-C**H**$_2$), 3.03 (t, ε-C**H**$_2$, t), 3.77 (t, α-C**H**)
myo-inositol	HMDB0000211, C00137	3.28 (t, 5-C**H**), 3.54 (dd, 1-C**H** & 3-C**H**), 3. 63 (t, 4-C**H** & 6-C**H**), 4.07 (t, 2-C**H**)
NAD$^+$	HMDB0000902, C00003	6.03 (d, 1'-C**H** ribose-adenine), 6.11 (d, 1'-C**H** ribose-nicotinamide), 8.18 (s, 2-C**H** adenine), 8.20 (m, 5-C**H** nicotinamide), 8.43 (s, 8-C**H** adenine), 8.84 (d, 4-C**H** nicotinamide), 9.15 (d, 6-C**H** nicotinamide), 9.34 (s, 2-C**H** nicotinamide)
Phenylalanine	HMDB0000159, C00079	7.34 (m, 2-C**H** & 6-C**H** ring), 7.39 (m, 4-C**H** ring), 7.43 (m, 3-C**H** & 5-C**H** ring)
Phosphocholine	HMDB0001565, C00588	3.23 (s, N(C**H**$_3$)$_3$), 3.60 (m, N-C**H**$_2$), 4.17 (m, HPO_4^- -C**H**$_2$)
Phosphocreatine	HMDB0001511, C02305	3.05 (s, N-C**H**$_3$), 3.95 (s, N-C**H**$_2$)
Proline	HMDB0000162, C00148	2.04 (m, γ-C**H**$_2$), 2.04/2.34(m/m, β-C**H**$_2$), 3.35/3.43 (dt/dt, δ-C**H**$_2$), 4.13 (dd, α-C**H**)
Pyruvate	HMDB0000243, C00022	2.38 (s, C**H**$_3$)

Succinate	HMDB0000254, C00042	2.41 (s, C**H**$_2$)
Taurine	HMDB0000251, C00245	3.27 (t, S-C**H**$_2$), 3.42 (t, N-C**H**$_2$)
Threonine	HMDB0000167, C00188	1.33 (d, *γ*-C**H**$_3$), 3.59 (d, *β*-C**H**), 4.25 (dd, *α*-C**H**)
Tyrosine	HMDB0000158, C00082	6.91 (d, 3-C**H** & 5-**H** ring), 7.20 (d, 2-C**H** & 6-**H** ring)
UDP-GalNAc	HMDB0000304, G10611	5.55 (dd, 1''-C**H** galactose)
UDP-GlcNAc	HMDB0000290, C00043	2.08 (s, C**H**$_3$ NAc), 3.82 (m, 3''-C**H** glucose & 6´´-C**H**$_2$ glucose), 3.88 (m, 6'' C**H**$_2$ glucose), 4.00 (m, 2''-C**H** glucose), 4.19/4.26 (m/m, 5'-C**H**$_2$ ribose), 4.30 (m, 4'-C**H** ribose), 4.38 (m, 2'-C**H** ribose & 3'-C**H** ribose), 5.52 (dd, 1''-C**H** glucose), 5.99 (m, 5-C**H** & 1'-C**H** ribose), 7.96 (d, 6-C**H**)
Valine	HMDB0000883, C00183	1.00 (d, *γ'*-C**H**$_3$), 1.05 (d, *γ*-C**H**$_3$), 2.27 (m, *β*-C**H**), 3.62 (d, *α*-C**H**)

Table S2

Metabolite	δ ^{1}H in ppm (multiplicity)[a]	Effect size (ES error %)/*p*-value	
		D_0 *vs.* D_{21} [b]	D_{0-7} *vs.* D_{14-21}
Amino acids			
Ala	1.48 (d)	-18.4(57.2)	-4.8(41.8)/0.0015 [c]
Gln	2.46 (m)	-5.5(63.7)	-4(44.3)/0.0015 [c]
Glu	2.35 (m)	-6.8(61.3)	
Gly	3.56 (s)	-3.8(70.3)	-3.4(46.9)/0.0015 [c]
Ile	1.02 (d)	-4(69.4)	-2.8(51.3)/0.0015 [c]
Leu	0.96 (d)	-5.5(63.7)	-2.6(53.4)/0.0032
Pro	2.04 (m)	-4.7(66.1)	-1.7(70.7)/0.0133
Tau	3.42 (t)	-15.3(57.5)	-3.2(48.5)/0.0022
Val	1.05 (d)	-4.9(65.2)	-1.7(72.1)/0.0095
Creatine	3.04 (s)		4(44)/0.0015 [c]
GSH	2.55 (m)		2.6(53)/0.0032
Choline derivatives			
Choline	3.21 (s)	14.9(57.6)	2.9(50.8)/0.0015 [c]
GPC	3.24 (s)	12.7(58)	3.1(48.7)/0.0015 [c]
Nucleotides and derivatives			
AMP	8.62 (s)		2.5(54.8)/0.0015 [c]
ADP	8.54 (s)		2.2(59.6)/0.0022
ATP	8.55 (s)	-8.8(59.4)	-2.1(61.8)/0.0015 [c]
UDP-GlcNAc	5.52 (dd)	4(69.1)	
1-MNA	4.49 (s)	-6.8(61.3)	-2.9(50.6)/0.0015 [c]
NAD^+	9.15 (d)		1.6(75)/0.025
Other metabolites			
Glucose	5.24 (d)	-6.5(61.7)	-4.6(42.3)/0.0015 [c]
Acetato	1.92 (s)	3.3(74.1)	
Ethanolamine	3.14 (t)		4.3(43.1)/0.0015 [c]
Unassigned compounds			
$U_{1.31}$	1.31 (br)	14.9(57.6)	2.1(60.4)/0.0095
$U_{1.41}$	1.41 (br)		-1.9(65.1)/0.0067
$U_{2.88}$	2.88 (s)		-3(49.3)/0.0015 [c]
$U_{3.48}$	3.48 (d)	10.3(58.7)	2(63.6)/0.0022
$U_{5.35}$	5.35 (br)		2.7(52.9)/0.0015 [c]
$U_{5.41}$	5.41 (br)		-1.7(71.2)/0.0184
$U_{7.68}$	7.68 (d)		1.7(70.2)/0.0184
